# The Role of Emotion in Psychosis Onset and Symptom Persistence: A Systematic Review

**DOI:** 10.1111/eip.70096

**Published:** 2025-10-04

**Authors:** R. Gurnani, A. Georgiades

**Affiliations:** ^1^ Department of Psychosis Studies Institute of Psychiatry, Psychology, and Neuroscience (IoPPN), King's College London London UK; ^2^ Croydon Talking Therapies, The New Building, Bethlem Royal Hospital Beckenham UK; ^3^ Brent Early Intervention Service, CNWL, NHS Foundation Trust London UK

**Keywords:** affect, cognitive model, emotion, emotion coping, psychosis

## Abstract

**Aim:**

Emotions have repeatedly been implicated in the development and maintenance of psychosis. However, there is no universally acknowledged theory to account for how emotions influence psychosis. This review, therefore, aims to explore how emotions and emotional coping strategies contribute to the onset and persistence of psychosis symptoms.

**Method:**

A systematic review was conducted to summarise the existing evidence base regarding the role of emotions and emotional coping strategies across the psychosis continuum, including individuals at Clinical High Risk (CHR), those experiencing a First Episode of Psychosis (FEP), and those with a diagnosis of Schizophrenia (SZ).

**Results:**

Seventy‐eight studies were eligible for inclusion. Compared to Healthy Controls (HCs), SZ and CHR individuals demonstrated significant impairments in emotional awareness, emotional understanding of self and others, and emotional regulation, along with heightened emotional reactivity. In SZ, lower emotional awareness, negative emotional reactivity, and emotional dysregulation were significantly associated with increased positive symptoms. Individuals with SZ reported high levels of Negative Affect (NA) and low levels of Positive Affect (PA), with NA being a strong predictor of paranoia and rumination strengthening the affective pathway to paranoia. In terms of coping, CHR and SZ demonstrated significantly greater use of Maladaptive Coping Strategies (MCS) than Adaptive Coping Strategies (ACS) compared to HCs. MCS such as suppression were significantly associated with increased positive and negative symptoms, social withdrawal, and depression severity in SZ, while ACS such as cognitive reappraisal significantly decreased negative symptoms, depression, and social withdrawal and improved social functioning. Individuals with SZ predominantly employed Emotion‐Focused Coping (EFC) rather than Problem‐Focused Coping (PFC), which were associated with negative and positive outcomes, respectively.

**Conclusion:**

These findings highlight the important role of emotion in psychosis onset and symptom persistence. Given the prominence of emotions in the manifestation and maintenance of psychosis, the development of emotion‐focused interventions for psychosis is necessary to not only prevent transition and relapse but also to maintain recovery. To support clinical application, this review also provides Socratic questions and recommends practical therapeutic tools to assist clinicians in the assessment, formulation, and intervention of emotional dysregulation in psychosis.

## 1 Introduction

The role of emotions has repeatedly been implicated in the development and maintenance of psychosis (Birchwood 2003; Birchwood and Trower 2006; Freeman and Garety 2003; Guillem et al. 2005; Häfner et al. 2005). Negative emotionality has been associated with an increased severity of psychosis symptoms, poor quality of life (QoL), reduced social functioning (an individual's interactions with their environment and their ability to fulfil their role in work, social activities, and relationships, Bosc 2000), greater cognitive impairment, and an increased risk of relapse (Booij et al. 2018; Chapman et al. 2020; Fialko et al. 2006; Kimhy et al. 2012; Liu et al. 2020; Myin‐Germeys et al. 2001). Key emotions in psychosis include anxiety, depression, guilt and shame, as well as feelings of low self‐esteem and hopelessness (Bentall and Fernyhough 2008; Birchwood et al. 2002; Freeman 2007; Garety et al. 2001). Anxiety and depression have been estimated to occur in approximately 60% and 28.6% of individuals with psychosis, respectively (Bosanac et al. 2016; Liu et al. 2020), with high anxiety being a significant predictor of psychosis emergence (Krabbendam et al. 2002). Specifically, guilt, shame, and anxiety have been implicated in delusion formation (Birchwood et al. 2002; Freeman 2007), with worry playing a causal role in the occurrence of persecutory delusions (Freeman et al. 2015), and depression and low self‐esteem contributing to auditory hallucination onset (Smith et al. 2006). Emotions have been hypothesised to contribute to the formation and maintenance of delusions, reflecting direct emotional concerns, while they are deemed less prominent in auditory hallucinatory content but may play a triggering role in its manifestation (Freeman and Garety 2003).

Psychosis has also been characterised by elevated stress reactivity (Myin‐Germeys and van Os 2007), heightened subjective emotional intensity (Kimhy et al. 2014), and a delayed return to emotional baseline levels (Vaessen et al. 2019; Ader et al. 2024). Compared to controls, individuals with a First Episode Psychosis (FEP) and those at Clinical High Risk (CHR) for psychosis who were exposed to high levels of emotional abuse exhibited prolonged negative affective recovery (Ader et al. 2024). Interestingly, positive affective recovery was not found to significantly differ between groups and was not associated with childhood trauma (Ader et al. 2024). Additionally, individuals with a FEP have demonstrated elevated rates of perceived stress (defined as the appraisal of an event as stressful, Cohen et al. 1983) accompanied by a lack of protective mechanisms to counteract the effects of stress by utilising Adaptive Coping Strategies (ACS) such as cognitive reappraisal and problem solving (Seitz et al. 2019). Instead, individuals with psychosis have frequently been found to use Maladaptive Coping Strategies (MCS), such as avoidance, rumination, and suppression (Ludwig, Mehl, Krkovic, and Lincoln 2020). Similarly, individuals at CHR have been found to rely more on MCS than ACS (Jalbrzikowski et al. 2014; Kang et al. 2018), suggesting that maladaptive coping is present during the at‐risk phase and may be relevant to the development of psychosis. Subsequently, there is growing evidence suggesting that poor coping responses to life stressors may play a more crucial role in the development, maintenance, and recovery of psychosis than the symptoms themselves (Myin‐Germeys and van Os 2007). Currently, there is no universally acknowledged theory to account for how emotions influence psychosis (Hartley et al. 2013). Understanding the role of emotions in psychosis onset and symptom persistence would aid in the development of a novel theoretical model for psychosis, which could facilitate personalised formulations and targeted interventions in Cognitive Behavioural Therapy for Psychosis (CBTp) practices. Therefore, a systematic review summarising the existing evidence base regarding emotions and emotional coping in psychosis is worthy of synthesis. To ensure clarity, definitions for key emotion‐related terms used throughout this review are provided in Table 1.

**TABLE 1:** Definitions for emotion terms.

Emotion term	Definition
Affect	The underlying experience of a feeling. Examples include a sense of pleasure or displeasure, tension or relaxation, and depression or elation (Russell and Barrett 1999)
Emotion	A specific form of affect, typically characterised by distinct experiences such as sadness, anxiety, anger, happiness, guilt, or shame and involves identifiable triggers, physiological responses, and behavioural expressions (Izard 2010)
Positive Affect	The experience of pleasurable emotions and feelings, such as joy, enthusiasm, and alertness (Watson et al. 1988)
Negative Affect	The experience of distress and negative emotional states such as sadness, anxiety, anger, guilt, shame, and disgust (Watson et al. 1988)
Emotional Functioning	One's expression, awareness, perception, and conceptualisation of emotions (Milojevich et al. 2021)
Emotional Awareness	The ability to recognise, identify, and understand one's own emotional states, as well as those of others (Lane and Schwartz 1987)
Emotional Reactivity	How quickly and intensely one experiences emotions in response to stimuli, encompassing both positive and negative emotional experiences (Vines et al. 2021)
Negative Emotional Reactivity	Significantly heightened increases in negative affect in response to everyday stress (Myin‐Germeys et al. 2003)
Emotional Regulation	The use of strategies to influence the duration, frequency, or intensity of a negative or positive emotion (Gross 1998)
Emotional Dysregulation	The inability to manage one's emotional responses, including the intensity, duration, and appropriateness of those responses (Cole et al. 1994)
Maladaptive Coping Strategies	Behaviours or thought patterns that may provide temporary relief but lead to negative health outcomes, such as avoidance, rumination, and suppression (Ludwig, Mehl, Krkovic, and Lincoln 2020)
Adaptive Coping Strategies	Behaviours or thought patterns that lead to positive health outcomes, such as reappraisal and distraction (Ludwig, Mehl, Krkovic, and Lincoln 2020)
Reappraisal	An emotion regulation strategy that involves reframing the meaning of a situation to alter its emotional impact (Gross 1998) It is an adaptive coping strategy where one changes their perspective regarding a situation in order to reduce negative emotions or to enhance positive ones
Acceptance	An adaptive coping strategy characterised by an open and welcoming approach towards emotions, thoughts, or external events (Hayes et al. 1999)
Savouring Positive Emotions	The focused attention on the present feelings of positive emotion (Bryant 2003) It is an adaptive coping strategy used to amplify pleasurable aspects of one's experience
Interpersonal Strategies	Talking about one's feelings with others (Strauss et al. 2019)
Avoidance	An individual's attempts to deny and minimise thoughts, feelings, and bodily experiences in response to negative emotions (Udachina et al. 2014)
Rumination	Perseverative thinking or dwelling on negative thoughts or feelings without seeking an active solution (Nolen‐Hoeksema et al. 2008)
Suppression	An emotion regulation strategy involving the conscious inhibition of emotionally expressive behaviour when emotionally aroused (Gross and Levenson 1993)
Distraction	The diversion of attention away from the emotion‐eliciting event (Grezellschak et al. 2015)
Situation Modification	Involves changing a situation to influence its emotional impact, typically by modifying the external environment or circumstances to reduce stress or enhance positive outcomes (Torrence and Connelly 2019)
Emotion‐Focused Coping	A strategy used to manage the emotional distress caused by a stressful situation, rather than addressing the situation itself. Strategies include seeking social support or social withdrawal, avoidance, practicing meditation, engaging in relaxation techniques, positive reframing (reframing the situation in a more positive light), and talking to others (Lazarus and Folkman 1984)
Problem‐Focused Coping	Strategies aimed at directly addressing or resolving the source of stress, with the goal of reducing or eliminating it (Lazarus and Folkman 1984)
Meta‐Emotional Beliefs	One's beliefs about emotions, including their nature, controllability, usefulness, and appropriateness. These beliefs influence how individuals understand, evaluate, and respond to their own emotional experiences (D'Amico and Geraci 2023)
Polyregulation	The use of several different strategies to regulate one's emotions during a given emotional scenario (Ladis et al. 2022)
Negative Affective Recovery	The return to one's emotional baseline following an increase in negative affect (Ader et al. 2024)
Positive Affective Recovery	The return to one's emotional baseline following a decrease in positive affect (Ader et al. 2024)
Ecological Momentary Assessment (EMA) Study	A random time‐sampling self‐report technique used to monitor emotions, behaviours, and symptoms in real‐world contexts via the use of mobile technology (Strauss et al. 2019)
Momentary in vivo use of ER strategies	The real‐time application of Emotion Regulation (ER) strategies during everyday life situations (Kimhy et al. 2020)
Low Emotional Granularity	The difficulty in differentiating between distinct emotional states (Kimhy et al. 2014)
Identification	Identifying the need to regulate one's emotional state (Gross 2015)
Selection	Identifying which strategy to select to regulate one's emotional state (Gross 2015 ^)^
Implementation	Implementing an emotional regulation strategy for a given context (Gross 2015)
Maintenance	The effective implementation of an emotional regulation strategy for a given context (Bartolomeo et al. 2022)

### 1.1 Aims

This systematic review aimed to summarise the existing literature regarding the role of emotion in psychosis onset and symptom persistence, as well as to identify adaptive and maladaptive emotional coping across the psychosis continuum, including individuals at CHR, those experiencing a FEP, and those with a diagnosis of Schizophrenia (SZ). Including CHR populations allows for the examination of emotional coping patterns in this at‐risk group and their potential contribution to the development of psychosis. The influence of childhood trauma on emotional processing will also be explored.

## 2 Method

The PRISMA guidelines (Page et al. 2021) were followed, and this study was registered with the PROSPERO Register (registration number: CRD42024551563).

### 2.1 Search Strategy

A systematic database search of MEDLINE (including PubMed), EMBASE, Global Health, and PsycINFO was conducted using an OVID search tool from database conception to 30th June 2024. The following search strings were used: Psychosis OR Schizophreni* OR Psychotic OR Clinical high risk OR Ultra high risk **AND** Emotion OR Emotion* processing OR Emotion* regulation OR Emotion* dysregulation OR Emotion* reactivity OR Affect* coping OR Affect* pathway OR Emotion* focused coping OR Emotion* oriented coping OR Suppress* **AND** Delusion* OR Hallucination* OR Positive symptom* OR Negative symptom* OR Psychotic symptom* **AND** Onset OR Maintenance OR Recover* OR Relaps* OR Function* OR Hospitali* OR Treatment adheren* OR Adheren* OR Quality of life OR Treatment response* OR Symptom severity OR Wellbeing.

### 2.2 Study Selection

**FIGURE 1:**
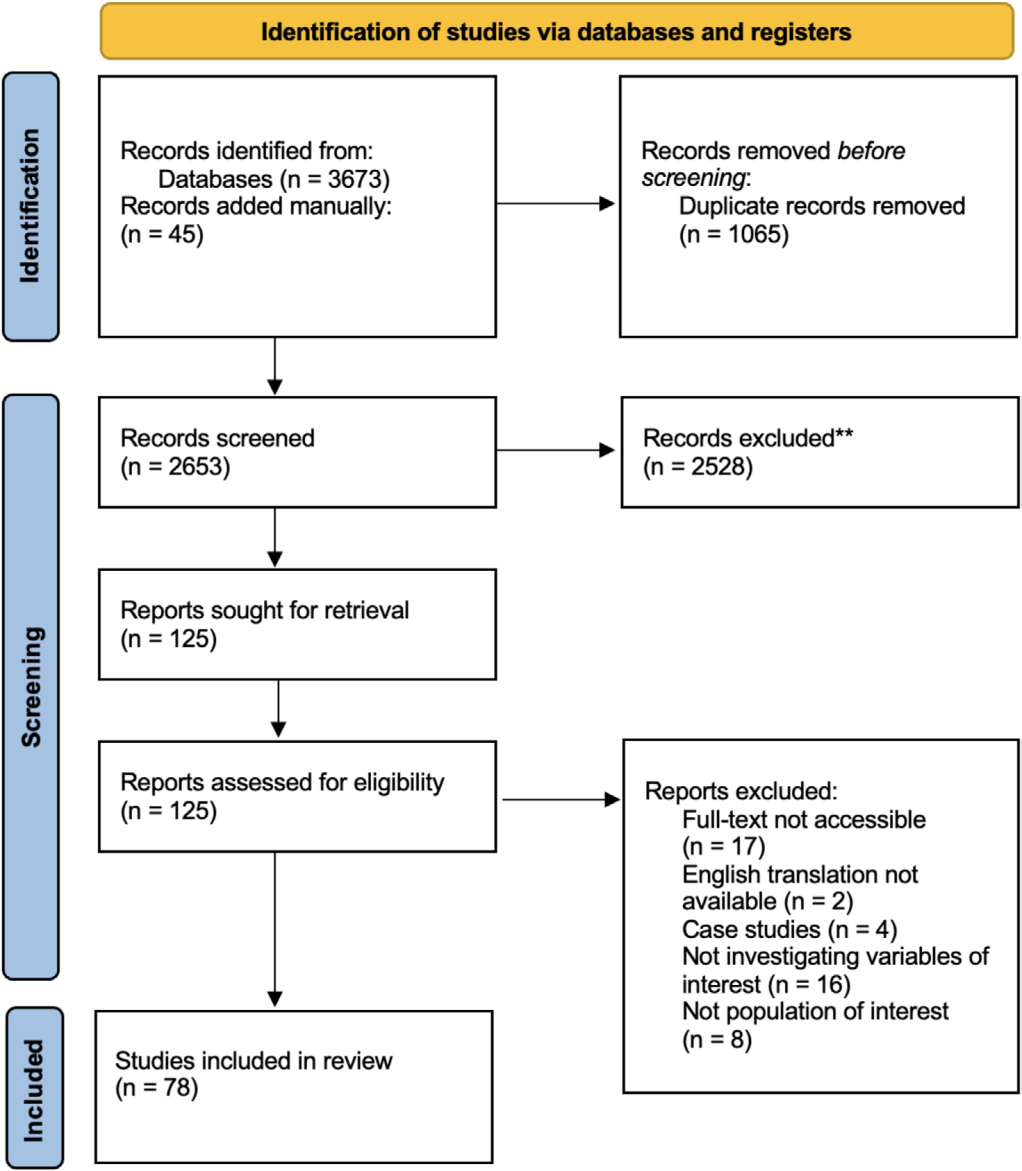
PRISMA 2020 flow diagram.

All studies, except for systematic reviews, were considered for inclusion. Eligible studies needed to (a) explore the role of emotions in CHR, FEP and/or SZ and/or (b) assess emotion coping strategies in CHR, FEP and/or SZ, and/or (c) assess whether interventions targeting emotion or emotion coping contributed to psychosis recovery. Eligibility required studies to be written in English, published in peer‐reviewed journals, and to include participants with a diagnosed psychotic disorder based on a validated diagnostic tool (e.g., ICD‐11; World Health Organization 2019; DSM‐5; American Psychiatric Association 2013), and/or individuals at CHR identified using established criteria or instruments such as the CAARMS (Yung et al. 2005), SIPS (Miller et al. 2003), or CAPE (Stefanis et al. 2002). The PRISMA flow diagram (see Figure 1) summarises the selection process.

### 2.3 Study Outcomes

The following variables of interest will be synthesised from the available literature.

**Emotional Functioning:** Refers to one's expression, awareness, perception and conceptualisation of emotions (Milojevich et al. 2021).

**Positive and Negative Affect:** Positive Affect (PA) refers to the experience of pleasurable emotions and feelings, such as joy, enthusiasm and alertness (Watson et al. 1988). In contrast, Negative Affect (NA) refers to the experience of distress and negative emotional states, such as sadness, anxiety, anger, guilt, shame and disgust (Watson et al. 1988).

**Maladaptive versus Adaptive Coping Strategies:** Maladaptive Coping Strategies (MCS) refers to behaviours or thought patterns that may provide temporary relief but lead to negative health outcomes, such as avoidance, rumination and suppression (Ludwig, Mehl, Krkovic, and Lincoln 2020). Adaptive Coping Strategies (ACS) refers to behaviours or thought patterns that lead to positive health outcomes, such as reappraisal and distraction (Ludwig, Mehl, Krkovic, and Lincoln 2020).

**Suppression versus Reappraisal:** Suppression is a form of emotional regulation defined as the conscious inhibition of emotionally expressive behaviour when emotionally aroused (Gross and Levenson 1993). Reappraisal is an emotion regulation strategy that involves reframing the meaning of a situation to alter its emotional impact (Gross 1998). It is an adaptive coping strategy where one changes their perspective regarding a situation in order to reduce negative emotions or to enhance positive ones.

**Emotion‐Focused Coping versus Problem‐Focused Coping:** Emotion‐Focused Coping (EFC) is a strategy used to manage the emotional distress caused by a stressful situation, rather than addressing the situation itself (Lazarus and Folkman 1984). EFC strategies include seeking social support or social withdrawal, avoidance, practicing meditation, engaging in relaxation techniques, positive reframing (reframing the situation in a more positive light), and talking to others (Lazarus and Folkman 1984). Problem‐Focused Coping (PFC) refers to strategies aimed at directly addressing or resolving the source of stress, with the goal of reducing or eliminating it (Lazarus and Folkman 1984).

**Anxiety, Depression and Positive Symptoms:** The role of anxiety and depression will be explored in relation to positive symptoms of psychosis such as auditory hallucinations and delusions.

**Interventions:** Interventions designed to improve emotional regulation in psychosis will be outlined.

**Qualitative Studies:** Studies that explore the subjective experience of emotion from individuals with psychosis will be summarised.

**Ecological Momentary Assessments (EMA) Studies:** An EMA is a random time‐sampling self‐report technique used to monitor emotions, behaviours and symptoms in real‐world contexts via the use of mobile technology (Strauss et al. 2019). EMA studies exploring the role of emotion and emotion coping strategies in psychosis will be discussed.

**Trauma and Emotional Vulnerability:** The influence of childhood trauma on emotional processing will also be outlined.

Although there is some conceptual overlap between sections (e.g., ‘maladaptive vs. adaptive coping’ and specific emotion regulation strategies such as ‘suppression vs. reappraisal’), this structure was retained to reflect how the original studies categorised and conceptualized emotional constructs, thereby allowing for a clearer distinction between broad coping styles and specific regulation strategies in psychosis.

### 2.4 Exclusion Criteria

Non‐English articles, systematic reviews/meta‐analyses, case studies, book chapters, conference abstracts and unpublished literature were excluded. Studies were also excluded if psychosis was due to substance misuse or if studies did not investigate an association between emotion or emotional coping and psychosis.

### 2.5 Data Extraction Process

The characteristics of studies were recorded within an excel spreadsheet after de‐duplication. The following data was extracted from the identified studies such as author and year, geographical location, study type, setting, sample characteristics, questionnaires, main results, and clinical findings. One reviewer (R.G.) conducted the screening for all papers, which was cross‐checked by a second reviewer (A.G.).

### 2.6 Quality Appraisal

The Effective Public Health Practice Project (EPHPP) (Thomas 2003) (see Table 2) and the Joanna Briggs Institute Critical Appraisal Checklist for Qualitative Research (Joanna Briggs Institute; Lockwood et al. 2015) (see Table 3) were employed to assess quality appraisals for quantitative and qualitative studies, respectively.

**TABLE 2:** Quality assessment for quantitative studies—EPHPP tool.

Author (year)	Selection bias	Study design	Confounders	Data collection methods	Withdrawals and dropouts	Analyses (appropriateness)	Global rating
Ader et al. (2024)	Moderate	Moderate	Weak	Strong	Weak	Yes	Weak
Bartolomeo et al. (2022)	Moderate	Moderate	Strong	Strong	Weak	Yes	Moderate
Beals et al. (2024)	Moderate	Moderate	Weak	Strong	Moderate	Yes	Moderate
Berglund et al. (2023)	Moderate	Moderate	Strong	Strong	Moderate	Yes	Strong
Dokuz et al. (2022)	Moderate	Moderate	Strong	Strong	Strong	Yes	Strong
Favrod et al. (2019)	Moderate	Moderate	Weak	Strong	Strong	Yes	Moderate
Foster et al. (2010)	Moderate	Strong	Weak	Strong	Strong	Yes	Moderate
Fung et al. (2023)	Moderate	Moderate	Strong	Strong	Weak	Yes	Moderate
Grove et al. (2016)	Moderate	Moderate	Strong	Strong	Moderate	Yes	Strong
Hartley et al. (2013)	Moderate	Moderate	Strong	Strong	Strong	Yes	Strong
Henry et al. (2008)	Moderate	Moderate	Strong	Strong	Moderate	Yes	Strong
Henry et al. (2008)	Moderate	Moderate	Strong	Strong	Moderate	Yes	Strong
Holubova et al. (2015)	Moderate	Moderate	Moderate	Strong	Moderate	Yes	Strong
Horan et al. (2013)	Moderate	Moderate	Weak	Strong	Moderate	Yes	Moderate
Jalbrzikowski et al. (2014)	Strong	Moderate	Strong	Strong	Strong	Yes	Strong
Kang et al. (2018)	Moderate	Moderate	Strong	Strong	Moderate	Yes	Strong
Kee et al. (2009)	Moderate	Moderate	Strong	Strong	Moderate	Yes	Strong
Kimhy et al. (2012)	Moderate	Moderate	Strong	Strong	Moderate	Yes	Strong
Kimhy et al. (2014)	Moderate	Moderate	Strong	Strong	Weak	Yes	Moderate
Kimhy et al. (2016)	Moderate	Moderate	Strong	Strong	Moderate	Yes	Strong
Kimhy et al. (2020)	Moderate	Moderate	Moderate	Strong	Weak	Yes	Moderate
Klippel et al. (2017)	Moderate	Moderate	Strong	Strong	Weak	Yes	Moderate
Klippel et al. (2022)	Moderate	Moderate	Strong	Strong	Weak	Yes	Moderate
Kommescher et al. (2014)	Moderate	Moderate	Strong	Moderate	Moderate	Yes	Strong
Kramer et al. (2013)	Moderate	Moderate	Strong	Strong	Strong	Yes	Strong
Krkovic et al. (2018)	Moderate	Moderate	Weak	Moderate	Weak	Yes	Weak
Lardinois et al. (2011)	Moderate	Moderate	Strong	Strong	Weak	Yes	Strong
Lawlor et al. (2022)	Moderate	Strong	Moderate	Strong	Moderate	Yes	Strong
Lee and Schepp (2010)	Moderate	Moderate	Weak	Moderate	Moderate	Yes	Moderate
Lehmann et al. (2014)	Moderate	Moderate	Strong	Strong	Moderate	Yes	Strong
Li et al. (2023)	Moderate	Strong	Strong	Strong	Strong	Yes	Strong
Li et al. (2024)	Moderate	Strong	Strong	Strong	Weak	Yes	Moderate
Lincoln et al. (2015a)	Moderate	Moderate	Moderate	Strong	Strong	Yes	Moderate
Lincoln et al. (2015b)	Moderate	Moderate	Moderate	Strong	Moderate	Yes	Moderate
Lincoln et al. (2017)	Moderate	Moderate	Moderate	Moderate	Moderate	Yes	Moderate
Liu et al. (2020)	Moderate	Moderate	Moderate	Moderate	Moderate	Yes	Strong
Livingstone et al. (2009)	Moderate	Moderate	Strong	Moderate	Moderate	Yes	Strong
Ludwig, Mehl, Krkovic, and Lincoln (2020)	Moderate	Moderate	Moderate	Moderate	Strong	Yes	Strong
Ludwig, Mehl, Schlier, et al. (2020)	Moderate	Moderate	Weak	Moderate	Strong	Yes	Moderate
Lysaker et al. (2011)	Moderate	Moderate	Strong	Strong	Weak	Yes	Moderate
Masillo et al. (2012)	Moderate	Moderate	Weak	Strong	Moderate	Yes	Moderate
Meyer (2001)	Moderate	Moderate	Moderate	Moderate	Moderate	Yes	Strong
Moran et al. (2018)	Moderate	Moderate	Strong	Moderate	Moderate	Yes	Strong
Moritz et al. (2016)	Moderate	Moderate	Moderate	Strong	Moderate	Yes	Strong
Myin‐Germeys et al. (2001)	Moderate	Moderate	Weak	Strong	Weak	Yes	Weak
Myin‐Germeys et al. (2003)	Moderate	Moderate	Weak	Strong	Strong	Yes	Moderate
Myin‐Germeys et al. (2005)	Moderate	Moderate	Moderate	Weak	Moderate	Yes	Moderate
Nittel et al. (2018)	Moderate	Strong	Strong	Strong	Moderate	Yes	Strong
Nowak et al. (2022)	Moderate	Moderate	Strong	Strong	Strong	Yes	Strong
Orth et al. (2022)	Moderate	Moderate	Strong	Strong	Moderate	Yes	Strong
Painter et al. (2019)	Moderate	Moderate	Strong	Weak	Moderate	Yes	Moderate
Palmier‐Claus et al. (2012)	Moderate	Moderate	Strong	Strong	Strong	Yes	Moderate
Parrish et al. (2023)	Moderate	Moderate	Moderate	Strong	Moderate	Yes	Moderate
Perry et al. (2011)	Moderate	Moderate	Weak	Strong	Moderate	Yes	Moderate
Piotrowski et al. (2019)	Moderate	Moderate	Weak	Strong	Moderate	Yes	Moderate
Raugh and Strauss (2022)	Moderate	Moderate	Moderate	Strong	Weak	Yes	Moderate
Raugh et al. (2025)	Moderate	Moderate	Moderate	Strong	Weak	Yes	Moderate
Reininghaus et al. (2016)	Moderate	Moderate	Strong	Strong	Weak	Yes	Moderate
Ritsner et al. (2006)	Moderate	Moderate	Moderate	Strong	Moderate	Yes	Strong
Rudnick (2001)	Moderate	Moderate	Moderate	Moderate	Moderate	Yes	Strong
Ryan et al. (2021)	Moderate	Moderate	Weak	Strong	Strong	Yes	Moderate
Smith et al. (2006)	Moderate	Moderate	Weak	Strong	Moderate	Yes	Moderate
Strakeljahn et al. (2023)	Strong	Moderate	Weak	Strong	Weak	Yes	Weak
Strauss et al. (2019)	Moderate	Moderate	Strong	Moderate	Moderate	Yes	Strong
Strauss et al. (2019)	Moderate	Moderate	Strong	Strong	Weak	Yes	Moderate
Strauss et al. (2023)	Moderate	Moderate	Strong	Strong	Weak	Yes	Moderate
Tarrier et al. (2007)	Moderate	Moderate	Weak	Strong	Moderate	Yes	Moderate
Thewissen et al. (2011)	Moderate	Moderate	Weak	Strong	Strong	Yes	Moderate
van der Steen et al. (2017)	Moderate	Moderate	Strong	Strong	Weak	Yes	Moderate
Vines et al. (2021)	Moderate	Moderate	Strong	Strong	Moderate	Yes	Strong
Visser et al. (2018)	Moderate	Moderate	Strong	Strong	Weak	Yes	Moderate
Yan Lam et al. (2020)	Moderate	Strong	Weak	Strong	Strong	Yes	Moderate
Yee et al. (2019)	Moderate	Moderate	Strong	Strong	Moderate	Yes	Strong
Zappia et al. (2012)	Moderate	Moderate	Moderate	Moderate	Moderate	Yes	Strong
Zarbo, Rota, et al. (2023)	Moderate	Moderate	Strong	Strong	Weak	Yes	Moderate
Zarbo, Zamparini, et al. (2023)	Moderate	Moderate	Strong	Strong	Strong	Yes	Strong

*Note:* EPHPP, Effective Public Health Practice Project (Thomas 2003).

**TABLE 3:** Quality assessment for qualitative studies—JBI tool.

Author (year)	1. Is there congruity between the stated philosophical perspective and the research methodology?	2. Is there congruity between the research methodology and the research question or objectives?	3. Is there congruity between the research methodology and the methods used to collect data?	4. Is there congruity between the research methodology and the representation and analysis of data?	5. Is there congruity between the research methodology and the interpretation of results?	8. Are participants, and their voices, adequately represented?	9. Is the research ethical according to current criteria or, for recent studies, and is there evidence of ethical approval by an appropriate body?	10. Do the conclusions drawn in the research report flow from the analysis, or interpretation, of the data?	Include if yes to 2–5, 8–10
Hutchins et al. (2016)	Yes	Yes	Yes	Yes	Yes	Yes	Yes	Yes	✓, ✓
Vodušek et al. (2014)	Yes	Yes	Yes	Yes	Yes	Yes	Yes	Yes	✓, ✓

*Note:* JBI, Joanna Briggs Institute Quality Assessment Tool (Lockwood et al. 2015).

## 3 Results

Of the 3673 papers identified, a total of 78 studies were included in this review. Table 4 summarises the findings of all studies eligible for this review.

**TABLE 4:** Characteristics of studies meeting inclusion criteria (*n* = 78).

Author (year)	Country and type of study	Sample size and setting	Mean age (SD)	Questionnaire and diagnostic tools	Main findings and clinical implications
Ader et al. (2024)	Belgium, Netherlands, UK Short‐Term Longitudinal Study (6 days)	113 FEP (49 F) 162 CHR (91 F) 94 HCs (34 M/60 F) Setting not specified	FEP 25.19 (5.14) CHR 23.48 (4.92) HCs 28.16 (5.47)	Ecological Momentary Assessment (EMA) Childhood Trauma Questionnaire (CTQ)	NA recovery (i.e., return to baseline following an increase in NA) was longer in individuals with first‐episode psychosis compared with HCs and in at‐risk individuals exposed to high versus low levels of emotional abuse. PA recovery (i.e., return to baseline following a decrease in PA) did not differ between groups and was not associated with childhood trauma. These results give first indications that NA recovery may be a putative momentary representation of resilience across stages of psychosis and may be amplified in at‐risk individuals with prior experiences of emotional abuse. Understanding how affective recovery contributes to the development of psychosis may help identify new targets for prevention and intervention to buffer risk or foster resilience in daily life.
Bartolomeo et al. (2022)	USA Short‐Term Longitudinal Study (6 days)	47 SZ (34% M/66% F) 52 HCs (30.8% M/69.2% F) Outpatient	SZ 39.53 (12.31) HCs 38.94 (10.26)	Ecological Momentary Assessment (EMA)	Results indicated that individuals with SZ exhibited excessive switching between emotion regulation strategies and delayed stopping compared to HCs, self‐efficacy moderated group differences in stopping abnormalities, and switching and stopping abnormalities were associated with different patterns of state‐level positive and negative symptoms in SZ. Findings may inform psychosocial emotion regulation therapies for SZ that could incorporate elements for monitoring dynamics and associated mechanisms.
Beals et al. (2024)	USA Cross‐sectional	44 SZ (32 M/12 F) 48 HCs (31 M/17 F) Outpatient	SZ 27.4 (5.6) HCs 30.1 (7.8)	Electronic Levels of Emotional Awareness Scale (eLEAS) Positive and Negative Syndrome Scale (PANSS) Childhood Trauma Questionnaire Short Form (CTQ‐SF)	SZ patients demonstrated significant deficits in emotional awareness overall, which was true for both self and others. In SZ patients, lower emotional awareness was significantly associated with more severe positive symptoms. Emotional awareness was significantly impaired in patients with self‐reported maltreatment exposure, relative to other groups. Severity of maltreatment was not significantly associated with emotional awareness or positive symptoms when looking continuously, and there was no significant indirect effect. These data suggest that emotional awareness impairments observed in SZ may be exacerbated by exposure to childhood maltreatment, possibly putting individuals at greater risk for experiencing positive symptoms of psychosis.
Berglund et al. (2023)	USA Cross‐sectional	72 SZ‐Spectrum (22.2% M/77.8% F) 61 HCs (21.3% M/78.7% F) Outpatient	SZ‐Spectrum 32.56 (12.92) HCs 33.76 (11.38)	Emotional Beliefs Questionnaire (EBQ) Emotion Regulation Questionnaire (ERQ) Brief Negative Symptom Scale (BNSS) Negative Symptom Inventory Self‐Report (NSI‐SR)	Those with SZ reported believing that emotions were less controllable than HCs; however, groups did not differ regarding beliefs about the usefulness of emotion. Greater beliefs of the uncontrollability of emotion were associated with greater use of suppression, less use of reappraisal, and increased negative symptoms. Emotion regulation partially mediated the association between emotional beliefs and negative symptoms. SZ display superordinate beliefs that emotions are uncontrollable. These beliefs may influence emotion regulation strategy selection and success, which contributes to negative symptoms. Findings suggest that beliefs of emotional uncontrollability reflect a novel process related to both emotion regulation and negative symptoms that could be targeted in psychosocial treatments.
Dokuz et al. (2022)	Turkey Cross‐sectional Short‐Term Longitudinal Study (6 days)	41 SZ (34 M/7 F) Outpatient	32.76 (8.79)	Ecological Momentary Assessment (EMA) Childhood Trauma Questionnaire (CTQ) Positive and Negative Syndrome Scale (PANSS)	Multilevel regression analyses showed that all trauma subtypes, except for sexual abuse, were associated with increased psychosis and event‐stress. Emotional maltreatment was the most associated trauma type with high negative and low PA and increased daily stress. Patients reported the highest stress and NA related to internal experiences, but the lowest stress related to recreational actions. Social activities were also associated with higher PA and lower stress and psychosis, with the high CTQ group having greater stress in those activities. This study demonstrates the negative impact of childhood trauma, especially emotional maltreatment, on daily stress and emotional and psychotic intensity in psychotic patients via different daily experiences.
Favrod et al. (2019)	France Cross‐sectional	21 SZ (16 M/5 F) Inpatient and Outpatient	33 (11.34)	Positive Emotions Program for SZ (PEPS) Scale for Assessment of Negative Symptoms (SANS) The Personal and Social Performance scale (PSP) Calgary Depression Scale for SZ (CDSS) Temporal Experience of Pleasure Scale (TEPS) Savouring Beliefs Inventory (SBI)	All SZ participants followed the 8 sessions of PEPS, and both composite scores were significantly and clinically improved at post‐test. Social functioning assessed with the PSP was also improved. This field test shows that participation in PEPS is accompanied by a reduction of negative symptoms and an improvement of social functioning. Both negative syndromes, reduction of expression and reduction of experience, are improved.
Foster et al. (2010)	UK Longitudinal	Total 24 SZ 12 Cognitive‐behavioural worry intervention (W‐CBT) (7 M/5 F) 12 Treatment‐as‐usual (7 M/5 F) Outpatient and Inpatient	Cognitive‐behavioural worry intervention (W‐CBT) 40.00 (10.00) 12 Treatment‐as‐usual 39.1 (9.2)	Penn State Worry Questionnaire (PSWQ) Psychotic Symptoms Rating Scale: Delusions Subscale (PSYRATS) Green et al. Paranoid Thought Scales (GPTS) Wechsler Test of Adult Reading (WTAR)	A cognitive‐behavioural worry intervention (W‐CBT) achieved a statistically significant reduction in worry which was maintained at 2 month follow up. A significant reduction in delusional distress was also reported. There was an indication that the worry intervention may also reduce the frequency of paranoid thoughts, but this was not statistically significant. In the first trial specifically for persecutory delusions, a brief worry intervention was shown to have benefits. The results support a causal role for worry in paranoid experience.
Fung et al. (2023)	Hong Kong Longitudinal	33 SZ (18 M/15 F) 35 HCs (19 M/16 F) Outpatient	SZ 25.0 (6.0) HCs 24.9 (5.7)	Ecological Momentary Assessment (EMA)	Adjusted multilevel linear‐mixed models revealed higher intensity and variability of NA in SZ than HCs, but no group difference in affect instability as well as PA intensity and variability. SZ demonstrated no significantly greater anhedonia for event, activity or social interactions relative to HCs. Higher preference for company (when alone) and to be alone (when in company) was observed in SZ than HCs. No significant group difference in pleasantness to be alone or proportion of time being alone. Results indicate no evidence for blunting of affective experiences, anhedonia (social and non‐social) and asociality in early psychosis.
Grove et al. (2016)	USA Cross‐sectional	277 SZ (150 M/127 F) 145 P‐NOS (78 M/67 F) 75 BD (39 M/56 F) 84 HCs (51 M/33 F) Outpatient	SZ 43.4 (11.2) P‐NOS 42.3 (10.9) BD 42.2 (11.2) HCs 42.0 (12.2)	Brief Assessment of Cognition in SZ (BACS) Mayer‐Salovey‐Caruso Emotional Intelligence Test (MSCEIT) Scale for the Assessment of Positive Symptoms (SAPS) Social Adjustment Scale—Self Report (SAS‐SR) Differential Emotions Scale (DES) Psychological Stress Index (PSI) The state subscale of the State–Trait Anxiety Inventory (Form X‐1; STAI‐S) The revised Beck Depression Inventory (BDI‐IA)	Elevated levels of NA were observed in clinical participants compared with HCs. For both clinical and HCs participants, NA measures were significantly correlated with social functioning, and consistently explained significant amounts of variance in functioning. For clinical participants, this relationship persisted even after accounting for cognition and positive/negative symptoms. The findings suggest that NA is a strong predictor of outcome across these populations and treatment of serious mental illnesses should target elevated NA in addition to cognition and positive/negative symptoms.
Hartley et al. (2013)	UK Short‐Term Longitudinal Study (6 days)	Total (22 M/11 F) 15 SZ 14 Psychosis not otherwise specified 2 Schizo‐affective disorder 1 Acute psychotic disorder	Total 33 (10.70)	Ecological Momentary Assessment (EMA) Positive and Negative Syndromes Scale (PANSS)	Antecedent worry and rumination predicted delusional and hallucinatory experience, and the distress they elicited. Using interaction terms, we have shown that the links with momentary symptom severity were moderated by participants' trait beliefs about worry/rumination, such that they were reduced when negative beliefs about worry/rumination (meta‐cognitions) were high. The findings offer an ecologically valid insight into the influence of worry and rumination on the experience of psychosis symptoms and highlight possible avenues for future intervention strategies.
Henry et al. (2008)	Australia Cross‐sectional	Low responders: 4 HCs (50% M/50% F) 12 SZ (33% M/777% F) High responders: 26 HCs (54% M/46% F) 17 SZ (53% M/47% F)	Low responders: HCs 35.5 (8.19) SZ 36.7 (11.1) High responders: HCs 34.6 (10.35) SZ 32.6 (7.64)	Wechsler's Abbreviated Scale of Intelligence (WASI) Film Clips	The results indicate that participants with SZ have difficulties with the amplification (but not suppression) of emotion expressive behaviour in response to video footage of 14 film clips selected from comedy shows and movies. These difficulties are significantly correlated with total negative symptoms experienced, particularly emotional blunting.
Henry et al. (2008)	Australia Cross‐sectional	41 SZ (46% M/54% F) 38 HCs (55% M/45% F) 36 Outpatients and 5 Inpatients Average Duration of Illness 14.6 years (10.50)	SZ 37.5 (10.67) HCs 36.1 (11.99)	The Scale for the Assessment of Positive Symptoms (SAPS) Wechsler's Abbreviated Scale of Intelligence (WASI) The Hospital Anxiety and Depression Scale (HADS) The Emotion Regulation Questionnaire (ERQ) The Social Functioning Scale (SFS)	Symptom severity and various aspects of cognitive and psychosocial functioning were also assessed. Relative to HCs, individuals with SZ did not differ with regard to their reported use of suppression and reappraisal, and reported use of both strategies was unrelated to clinical ratings of blunted affect. However, whereas (lower) use of reappraisal was associated with greater social function impairment for both groups, only for HCs was (greater) use of suppression associated with reduced social functioning. Implications for understanding blunted affect and social dysfunction in SZ are discussed.
Holubova et al. (2015)	Slovakia Cross‐sectional	103 SZ (41 M/62 F) Outpatient	41.96 (10.23)	Stress Coping Style Questionnaire (SVF‐78) Quality of Life Satisfaction and Enjoyment Questionnaire (Q‐LES‐Q) Clinical Global Impression (CGI)	QoL was significantly related to both the positive and negative coping strategies. The severity of disorder was highly negatively correlated with the QoL score. The results of multiple stepwise regression analysis using the quality of life as a dependent variable showed that symptom severity, negative coping strategies, positive coping strategies, and the difference between positive and negative coping strategies explain more than half variance. This study suggests the importance of utilising the positive coping strategies in improving the quality of life in patients with psychotic disorders.
Horan et al. (2013)	USA Cross‐sectional	31 SZ (75% M/25% F) 27 HCs (77.4% M/22.6% F) Outpatient	SZ 47.8 (9.8) HCs 45.5 (6.7)	Brief Psychiatric Rating Scale (BPRS) Emotion Regulation Questionnaire (ERQ) Event‐Related Potentials (ERPs)	HCs showed a clear pattern where the Late Positive Potential (LPP) was significantly reduced when unpleasant pictures were described in less negative or more neutral terms before being shown. Specifically, in the Neutral Description condition, their LPP did not differ from the Neutral condition during the earliest time window (300–600 ms) and fell between the Neutral and Negative Description conditions during the middle two time windows (601–1500 ms). In contrast, SZ patients were unable to reduce their LPP responses. They showed (a) higher LPPs for both description conditions compared to the Neutral condition in the early time window and (b) an opposite pattern to HCs in the middle time windows, with larger LPPs for the Neutral Description condition than for the Negative Description and Neutral conditions. Additionally, SZ patients reported using cognitive reappraisal less often and expressive suppression more often than HCs.
Hutchins et al. (2016)	UK Qualitative study	8 FEP (6 M/2 F) Duration of illness not reported Early Intervention Service	Age range 19–35 years (MD and SD not specified)	Semi‐structured interviews	Four themes were generated by the analysis. The first highlighted emotional experiences prior to the onset of psychosis: ‘struggling with life distress’. The second highlighted the intense emotional experience within psychotic experiences: ‘transformed world and intense emotion’. The third theme highlighted self‐critical tendencies in the post‐onset phase of psychosis: ‘blame and guilt after the breakdown’. The final theme highlighted a mixture of emotions in the post‐onset phase: ‘confusion, despair and hope’. These findings highlight the value of normalising participants' emotional experiences in order to promote engagement in services and of assessing for self‐criticism, despair and hope following the psychotic experience, alongside therapeutically addressing the varying levels of emotional experiences before, during and after a psychotic breakdown.
Jalbrzikowski et al. (2014)	USA Longitudinal	88 CHR (58 M/30 F) 53 HCs (27 M/26 F) Setting not specified	CHR 17.9 (3.4) HCs 17.8 (4.4)	Global Functioning Social (GSF) Global Functioning Role (GRF) Wechsler Abbreviated Scale of Intelligence (WASI) Brief Cope Questionnaire Structured Interview for Prodromal Symptoms (SIPS)	Cross‐sectional analyses revealed that, in comparison to HCs, CHR youth reported using more maladaptive coping strategies and fewer adaptive coping strategies. Longitudinal analyses within the CHR group showed significant decreases in maladaptive coping and symptom severity over time, with corresponding improvements in social and role functioning. Adaptive coping was associated with better concurrent social functioning and less severe symptomatology (both *p* < 0.001). Over time, more maladaptive coping was associated with more severe positive and negative symptoms. Youth at‐risk for psychosis report using fewer adaptive and more maladaptive coping strategies relative to HCs. Over 1 year follow‐up, more adaptive coping styles are associated with less severe clinical symptomatology and better social functioning. These findings suggest that teaching adaptive coping styles may be an important target for intervention in youth at high risk for psychosis.
Kang et al. (2018)	South Korea Cross‐sectional	65 UHR (39 M/26 F) 83 HCs (41 M/42 F) Setting not specified	UHR 20.1 (3.4) HCs 20.8 (3.6)	The Korean version of Ways of Coping Questionnaire (K‐WCQ) The Ambiguous Intentions Hostility Questionnaire (AIHQ) Korean‐translated version of the Interpersonal Support Evaluation List Self‐efficacy Scale Wechsler Adult Intelligence Scale	UHR participants relied more on a passive, tension‐reduction coping style and less on an active, problem‐focused coping style. These maladaptive coping styles in UHR individuals were significantly associated with their cognitive appraisals of stress. Aberrant attribution style of hostility perception and composite blaming bias were associated with problem‐focused coping and tension‐reduction, respectively. Perceived social support was related to problem‐focused coping, seeking social support, and wishful thinking. General self‐efficacy was associated with problem‐focused coping. These findings suggest that cognitive appraisals themselves may be the major determinants of coping styles in UHR individuals. The identified attribution styles, perceived social support, and self‐efficacy may provide some clues regarding specialised interventions for the buildup of adaptive coping strategies in UHR individuals.
Kee et al. (2009)	USA Cross‐sectional	50 SZ (62% M/38% F) 39 HCs (65% M/35% F) Outpatient	SZ 34.37 (7.69) HCs 32.97 (5.17)	Mayer Salovey Caruso Emotional Intelligence Test (MSCEIT) Scale for the Assessment of Negative Symptoms (SANS) Role Functioning Scale (RFS) UCLA Social Attainment Scale (SAS)	The MSCEIT demonstrated good psychometric properties in both groups. SZ performed significantly worse than HCs on the total MSCEIT score, and on three of the four subtests: Identifying, Understanding, and Managing Emotions. Among SZ, lower MSCEIT scores significantly correlated with higher negative and disorganised symptoms, as well as worse community functioning. The MSCEIT is a useful tool for investigating emotion processing in SZ. Individuals with SZ demonstrate deficits across multiple domains of emotion processing. These deficits have significant links with clinical symptoms of SZ and with how SZ function in their daily lives. Further research is required to understand the links between emotional intelligence, clinical symptoms, and functional outcome in SZ.
Kimhy et al. (2012)	USA Cross‐sectional	44 SZ (64% M/36% F) 20 HCs (50% M/50% F) Inpatient and Outpatient	SZ 30.33 (8.08) HCs 24.20 (4.62)	Toronto Alexithymia Scale (TAS‐20) Emotion Management Task (EMT) The Emotion Regulation Questionnaire (ERQ) Provision of Social Relations Scale (PSRS) Self‐Reflection index of the Beck Cognitive Insight Scale (BCIS‐SR) MATRICS Consensus Cognitive Battery (MCCB) Mayer Salovey Caruso Emotional Intelligence Test (MSCEIT)	Compared to HCs, individuals with SZ displayed significant deficits describing and identifying their emotions and used significantly less reappraisal and more suppression to regulate their emotions. Among the SZ group, better social functioning was associated with the ability to identify, and in particular to describe emotions, better emotion management, as well as greater use of reappraisal and less use of suppression. A hierarchical multiple regression analysis indicated that, after controlling for age and neurocognition, difficulties describing feelings accounted for 35% of the social functioning variance. This study highlights the importance of emotion awareness and regulation in SZ, pointing to their substantial influence on social functioning above and beyond the impact of neurocognitive functioning.
Kimhy et al. (2014)	USA Short‐Term Longitudinal Study (2 days)	77 SZ (45 M/32 F) 27 HCs (10 M/17 F) Inpatient and Outpatient	SZ 32.51 (9.19) HCs 23.95 (5.01)	Ecological Momentary Assessment (EMA) Provision of Social Relations Scale (PSRS) Mayer Salovey Caruso Emotional Intelligence Test (MSCEIT) Scale for Assessment of Negative Symptoms (SANS) Toronto Alexithymia Scale (TAS‐20) Wechsler Test of Adult Reading (WTAR) Beck Depression Inventory (BDI) Beck Anxiety Inventory (BAI) Scale for Assessment of Positive Symptoms (SAPS) MATRICS Consensus Cognitive Battery (MCCB)	Compared to HCs, individuals with SZ displayed significantly poorer social functioning and lower on all emotional granularity indices (EGIall), but comparable on the index for negative emotions (EGIneg). Within the SZ group, hierarchical multiple regression analyses indicated that EGIall, but not EGIneg, significantly predicted social dysfunction after controlling for emotional awareness, symptoms, and emotional intensity and variability. These findings indicate that individuals with SZ have a relatively intact ability to differentiate among negative emotions in everyday life. However, they experience significant difficulties differentiating between positive and negative emotions, and this may contribute to their social difficulties.
Kimhy et al. (2016)	USA Cross‐sectional	54 CHR (74% M/26% F) 87 SZ (63% M/37% F) 50 HCs (52% M/48% F) Inpatient and Outpatient	CHR 20.18 (3.41) SZ 33.45 (9.47) HCs 23.04 (4.10)	Toronto Alexithymia Scale (TAS‐20) Wechsler Abbreviated Scale of Intelligence (WASI) Beck Depression Inventory (BDI) Beck Anxiety Inventory (BAI) Emotion Regulation Questionnaire (ERQ) Global Functioning Scale: Social (GFS:S) Structured Interview for Prodromal Symptoms (SIPS)	Group comparisons indicated significant differences between HCs and the two clinical groups in their ability to identify and describe feelings, as well as the use of suppression and reappraisal emotion‐regulation strategies. Specifically, the CHR and SZ groups displayed comparable deficits in all domains of emotion awareness and emotion regulation. A hierarchical multiple regression analysis indicated that difficulties describing feelings accounted for 23.2% of the SF variance. These results indicate that CHR individuals display substantial emotion awareness and emotion‐regulation deficits, at severity comparable with those observed in individuals with SZ. Such deficits, in particular difficulties describing feelings, predate the onset of psychosis and contribute significantly to poor SF in this population.
Kimhy et al. (2020)	USA Short‐Term Longitudinal Study (36‐h period)	54 SZ (40% M/60%) Inpatient and Outpatient	32.31 (8.23)	Ecological Momentary Assessment (EMA) Scale for the Assessment of Positive Symptoms (SAPS) Diagnostic Interview for Genetic Studies (DIGS) Toronto Alexithymia Scale (TAS‐20) Emotion Regulation Questionnaire (ERQ)	Use of suppression predicted significant increases in momentary experiences of thought insertion, mind reading, auditory and visual hallucinations. Use of reappraisal predicted significant increases in momentary experiences of suspiciousness, thought insertion, and mind reading. Emotion awareness, driven primarily by difficulties identifying feelings, moderated the impact of ER on psychotic symptoms. There were no associations between retrospective measures of ER and symptoms. These results indicate that, among individuals with SZ, emotion awareness significantly impacts the relationship between use of ER and exacerbations in psychotic symptoms during the course of daily functioning. Further, these results highlight the need to incorporate emotion awareness and regulation difficulties into the development of treatment models and interventions for psychosis. In addition, our results underscore the need to employ in vivo, high time‐resolution assessment methods to study dynamic clinical phenomena such as ER and psychotic symptoms.
Klippel et al. (2017)	UK Short‐Term Longitudinal Study (6 days)	51 FEP (28 M/23 F) 46 ARMS (21 M/25 F) 53 HCs (25 M/28 F) Setting not specified	FEP 28.3 (8.6) ARMS 23.6 (4.7) HCs 35.0 (12.6)	Ecological Momentary Assessment (EMA)	The effects of stress on psychotic experiences were mediated via pathways through affective disturbance in all three groups. The effect of stress on psychotic experiences was mediated by threat anticipation in FEP individuals and HCs but not in ARMS individuals. There was only weak evidence of mediation via aberrant salience. However, aberrant salience retained a substantial direct effect on psychotic experiences, independently of stress, in all three groups. These provide novel insights on the role of affective disturbance and threat anticipation in pathways through which stress impacts on the formation of psychotic experiences across different stages of early psychosis in daily life.
Klippel et al. (2022)	Belgium, Netherlands, Germany, UK Short‐Term Longitudinal Study (6 days)	245 PD (111 M/132 F) 165 Unaffected First‐Degree Relatives (68 M/97 F) 244 HCs (111 M/132 F)	PD 35.3 (10.8) FDR 36.7 (12.7) HCs 36.4 (12.4)	Ecological Momentary Assessment (EMA)	Evidence on indirect effects from cross‐sectional models indicated that, in all three groups, effects of stress on psychotic experiences were mediated by NA and, vice versa, effects of psychotic experiences on stress were mediated by NA, with all indirect effects being weakest in relatives. Longitudinal modelling of data provided no evidence of temporal priority of stress in exerting its indirect effects on psychotic experiences via affective disturbance or, vice versa. These findings tentatively suggest a rapid vicious cycle of stress impacting psychotic experiences via affective disturbances, which does, however, not seem to be consistently modified by familial liability to psychosis.
Kommescher et al. (2014)	Germany Cross‐sectional	Total 91 CHR (57 M/34 F) 45 Integrated Psychological Intervention (IPI) (30 M/15 F) 46 Supportive Counselling (SC) (27 M/19 F) Outpatient	IPI 24.1 (4.3) SC 26.9 (6.0)	Stress Coping Questionnaire (SCQ 120) Positive and Negative Syndromes Scale (PANNS) Montgomery Åsberg Depression Scale (MADRS) Global Assessment of Functioning Scale (GAF)	CHR participants relied significantly more on negative than on positive coping strategies, and within the positive strategies, stress control was the most preferred one. Several pre‐therapy coping strategies significantly predicted improvement in symptomatic outcome in both treatment groups, explaining between 16% and 25% of variance. The predictive value of coping was higher in the SC group. These findings suggest that maladaptive coping behaviours were found to emerge in the early stages of psychosis and coping behaviour contributed significantly to the prediction of post‐ treatment symptom improvement. These findings indicate a need for psychosocial support and coping strategy enhancement in people at risk of psychosis.
Kramer et al. (2013)	Belgium Cross‐sectional Short‐Term Longitudinal Study (5 days)	515 (All female) General population sample Outpatient	27.7 (8.3)	Ecological Momentary Assessment (EMA) Childhood Trauma Questionnaire (CTQ) Community Assessment of Psychic Experiences (CAPE) Symptom Checklist‐90‐Revised (SCL‐90‐R)	Moments of NA increase resulted in a significant increase in paranoia over 180 subsequent minutes. Both stress sensitivity and depressive symptoms impacted on the transfer of NA to paranoia. Stress sensitivity moderated the level of increase in paranoia during the initial NA increase, while depressive symptoms increased persistence of paranoid feelings from moment to moment. Momentary paranoia responses to NA increases were associated with follow‐up psychotic symptoms. Examination of microlevel momentary experience may thus yield new insights into the mechanism underlying co‐occurrence of altered mood states and psychosis. Knowledge of the underlying mechanism is required in order to determine source and place where remediation should occur.
Krkovic et al. (2018)	Germany Cross‐sectional Short‐Term Longitudinal Study (24 h)	67 CHR (28.4% M/71.6 F%)	23.01 (4.63)	Ecological Momentary Assessment (EMA) Community Assessment of Psychic Experiences (CAPE) Trauma History Questionnaire (THQ) Community Assessment for Psychic Experiences (CAPE)	Multilevel analysis showed that NA (*p* < 0.001) and heart rate (*p* < 0.05) were predictive of subsequent threat beliefs. There was no significant mediation effect from any trauma characteristic to threat beliefs via NA and heart rate. Trauma frequency (*p* < 0.001), age at first trauma (*p* < 0.001), as well as the presence of physical trauma (*p* < 0.001) moderated the path from NA to subsequent threat beliefs. The findings indicate that more frequent trauma, trauma at young age and physical trauma strengthen the association from NA to threat beliefs and could be relevant to determining the extent of vulnerability to psychosis.
Lardinois et al. (2011)	Netherlands, Belgium Short‐Term Longitudinal Study (6 days) Cross‐sectional	50 Psychosis (35 M/15 F) Outpatient and Inpatient	26.2 (6.7)	Ecological Momentary Assessment (EMA) Childhood Trauma Questionnaire (CTQ)	A significant interaction was found between stress and CT on both NA (event stress: *b* = 0.04, *p* < 0.04; activity stress: *b* = 0.12, *p* < 0.001) and psychotic intensity (event stress: *b* = 0.06, *p* < 0.001; activity stress: *b* = 0.11, *p* < 0.001), showing that a history of CT is associated with increased sensitivity to stress. A history of childhood trauma in patients with psychosis is associated with increased stress reactivity later in life, suggestive for an underlying process of behavioural sensitization.
Lawlor et al. (2022)	UK Longitudinal Pre‐Post DBT Group Therapy 8 sessions (90 min weekly)	Total 48 (22 M/26 F) 35 SZ 6 BD 4 PDD 2 UNP 1 ATPD 1 ONPD Outpatient	43 (11)	Clinical Outcomes in Routine Evaluation Outcome Measure (CORE‐10) Difficulties in Emotion Regulation Scale‐16 (DERS‐16) DBT Skills Subscale (DSS) The Managing Emotions Group Intervention (MEG)	Forty‐eight SZ participants (81% of attenders) completed the intervention (M age = 43, 54% female) of whom 39 completed pre‐ and post‐group measures. Participants reported high satisfaction and meaningful improvements in understanding and managing emotions, with positive impact on daily life. Self‐reported psychological distress, ER difficulties, and adaptive ER skill use significantly improved, with medium‐to‐large pre‐post effects (*d* = 0.5–0.7) except lack of emotional clarity (*d* = 0.3). These findings suggest that the Managing Emotions Group was feasible and acceptable, and a future feasibility randomised controlled trial is warranted.
Lee and Schepp (2010)	USA Cross‐sectional	40 SZ (32 M/8 F) 30 HCs (13 M/17 F) Setting not specified	SZ 17.25 (1.37) HCs 17.10 (1.16)	Ways of Coping (WOC) Symptoms of Stress (SOS) Personal Control Scale Amount of Support Scale: Teacher Version (ASS:T)	Adolescents with SZ used emotion‐focused coping more than problem‐focused coping at baseline and 6 weeks (*p* < 0.01). Subjects reported higher stress than HCs (*t* = 4.73, *p* < 0.01) and used emotion‐focused coping with emotional stress responses (*r* = 0.34, *p* = 0.05). These findings suggest that adolescent coping strategies may persist into adulthood unless new skills are introduced. Developing effective coping skills for adolescents with SZ is important for practice and future studies.
Lehmann et al. (2014)	Germany Cross‐sectional	55 SZ (32 M/23 F) 55 HCs (30 M/25 F) Inpatient and Outpatient	SZ 39.8 (11.9) HCs 38.9 (12.8)	The Multifaceted Empathy Test (MET) The Interpersonal Reactivity Index (IRI) The Emotional Contagion Scale (ECS) The Subjective Experience of Emotions Scale (SEE)	Individuals with SZ showed impairments of cognitive empathy, but maintained emotional empathy. They reported significantly more negative emotional contagion, overwhelming emotions, lack of emotions, and symbolization of emotions by imagination, but less self‐ control of emotional expression than HCs. Besides cognitive empathy, the experience of a higher extent of overwhelming emotions and of less interpersonal distress predicted psychosocial function in patients. People with SZ and HCs showed diverging patterns of how cognitive and emotional empathy related to the subjective aspects of emotion processing. It can be assumed that variables of emotion processing are important moderators of empathic abilities in SZ.
Li et al. (2023)	Belgium, Netherlands Short‐Term Longitudinal EMA study (6 days)	68 UHR (29 M/39 F) 55 FEP (29 M/26 F) Setting not specified	UHR 24.26 (5.81) FEP 25.76 (6.34)	Ecological Momentary Assessment (EMA) Brief Psychiatric Rating Scale (BPRS)	Multilevel mixed effect models showed that intensity appraisal was most closely associated with ER strategy use, as opposed to importance and controllability appraisals. Higher intense negative events were associated with more rumination and social sharing, while less intense negative events were associated with more reappraisal. Higher intense positive events were associated with a greater number of deployed strategies and more efforts in using savouring, expression and social sharing. The UHR and FEP individuals did not significantly differ regarding effects of above‐mentioned appraisal dimensions on ER. These results provide evidence supporting ER flexibility in early psychosis, and event intensity emerged as the dimension most strongly associated with ER.
Li et al. (2024)	Belgium, Netherlands Short‐Term Longitudinal EMA study (6 days)	67 CHR 52 FEP Total (55 M/64 F) Setting not specified	Total 24.76 (5.91)	Ecological Momentary Assessment (EMA) Comprehensive Assessment of At Risk Mental State (CAARMS)	Multilevel models of within‐person associations showed that greater ER flexibility was associated with more stable NA, and quicker recovery of NA from stressors during the day. Linear regression analyses of between‐person associations showed that people who had more variable and unstable NA reported greater ER flexibility generally. No evidence was found for associations with NA inertia. The study identified unique within‐person and between‐person links between ER flexibility and dynamics of NA in early psychosis. These findings further provide evidence for ER flexibility in early psychosis, emphasising the adaptive nature of regulatory flexibility in relation to reduced instability in NA and faster recovery from NA in everyday life.
Lincoln et al. (2015a)	Germany Cross‐sectional	35 SZ (21% M/14% F) 28 HCs (16% M/12% F) Inpatient and Outpatient	SZ 40.5 (12.5) HCs 35.6 (14.5)	Positive and Negative Syndrome Scale for Schizophrenia (PANSS) Emotion‐Specific Emotion Regulation Skills Questionnaire (ERSQ‐ES)	Participants with psychosis showed a stronger increase in self‐reported stress and Skin Conductance Levels (SCL) in response to the stressor than HCs. Stronger increases in self‐reported stress were predicted by a reduced ability to be aware of and tolerate distressing emotions, whereas increases in SCL were predicted by a reduced ability to be aware of, tolerate, accept and modify them. Although paranoid symptoms were not significantly affected by the stressors, individual variation in paranoid responses was also predicted by a reduced ability to be aware of and tolerate emotions. Differences in stress responses in the samples were no longer significant after controlling for ER skills. Thus, interventions that improve ER‐skills could reduce stress‐sensitivity in psychosis.
Lincoln et al. (2015b)	Germany Cross‐sectional	37 PD (57 M/43 F) 30 Depression (53 M/47 F) 28 HCs (57 M/43 F) Inpatient and Outpatient	PD 40.3 (12.3) Depression 41.7 (11.1) HCs 35.6 (14.5)	Positive and Negative Syndrome Scale (PANSS) Community Assessment of Psychic Experiences (CAPE) Emotion‐Specific Emotion Regulation Skills Questionnaire (ERSQ‐ES)	Compared with HCs, participants with PD showed reduced skills related to awareness, understanding and acceptance of potentially distressing emotions, but not in the ability to modify them. These differences remained significant after controlling for depression. Participants with PD showed reduced ER skills in regard to all of the assessed emotions compared with HCs, despite the fact that they only reported sadness as being significantly more intense. The participants with depression showed a similar pattern of ER skills to the PD sample, although with a tendency towards even more pronounced difficulties. It is concluded that PD is characterised by difficulties in using specific ER skills related to awareness, understanding and acceptance to regulate anger, shame, anxiety and sadness. These difficulties are not unique to PD but nevertheless present a promising treatment target.
Lincoln et al. (2017)	Germany, Indonesia, and USA Longitudinal	562 PD (285 M/277 F) Outpatient	35.99 (12.77)	Community Assessment of Psychic Experiences (CAPE) Emotion Regulation Skills Questionnaire (ERSQ)	The cross‐lagged paths from impaired ER to symptom distress (but not frequency) were significant. However, there was also evidence for the reverse causation from symptom frequency and distress to impaired ER. ER partially mediated the significant prospective paths from childhood trauma to symptom distress. The findings demonstrate that ER plays a role in translating childhood trauma into distressing psychotic experiences in later life. Moreover, the findings point to a maintenance mechanism in which difficulties in ER and symptom distress exacerbate each other. Thus, ER could be a promising target for interventions aimed at prevention of psychosis.
Liu et al. (2020)	Singapore Cross‐sectional	150 Non‐affective PD (85 M/65 F) Outpatient	26.5 (6.20)	Stressful Life Events Questionnaire (SLESQ) PTSD Checklist for DSM‐5 (PCL‐5) Brief Psychiatric Rating Scale‐Expanded (BPRS‐E) Cognitive Emotion Regulation Questionnaire Short (CERQ) Difficulties with Emotion Regulation Scale Short (DERS)	Mediation analyses controlling for gender, psychiatric comorbidities, antipsychotic medication dosage, duration of untreated psychosis (DUP), family history of mental illness, and cumulative trauma revealed that maladaptive CERS (rumination, catastrophic thinking, and self‐blame) and global emotion dysregulation mediated the effects of probable PTSD on depressive symptoms (*R* ^2^ = 41%), while maladaptive CERS, global emotion dysregulation, and depressive symptoms mediated the effects of probable PTSD on positive symptoms (*R* ^2^ = 30%). These results demonstrate the indirect effects of maladaptive CERS and global emotion dysregulation on maintaining depressive and positive symptoms. Emotion dysregulation may be a potential transdiagnostic treatment target to alleviate depressive and positive symptoms in traumatised patients with early non‐affective psychosis.
Livingstone et al. (2009)	UK Cross‐sectional	21 PD (12 M/9 F) 21 Anxiety/Mood disorder (5 M/16 F) 21 HCs (12 M/9 F) Outpatient	PD 39.26 (11.30) Anxiety/Mood disorder 40.52 (10.67) HCs 40.00 (11.88)	The Emotion Regulation Questionnaire (ERQ) The Regulation of Emotions Questionnaire (REQ) The Basics Emotions Scale (BES)	Both clinical groups were found to experience similar levels of emotions, and in comparison to HCs, they experienced greater levels of negatively valenced emotions and lower levels of happiness. Both clinical groups also used similar emotion regulation strategies, and in comparison to HCs, they used significantly more dysfunctional and less functional strategies, suggesting that the emotional experience and emotion regulation strategies of people who have experienced psychosis are more similar to non‐PDs than have previously been thought to be the case.
Ludwig, Mehl, Krkovic, and Lincoln (2020)	Germany Short‐Term Longitudinal EMA study (6 days)	71 PD (57.8% M/42.2% F) Outpatient	37.80 (12.15)	Ecological Momentary Assessment (EMA) EMA used to assess NA and ER strategies Paranoia Checklist Positive and Negative Syndrome Scale (PANSS) Calgary Rating Scale for SZ (CDSS) Role Functioning Scale (RFS)	Multilevel regression analysis revealed that higher awareness at t‐1 reduced the association of NA at t‐1 and paranoia at t, whereas rumination had an opposite, amplifying moderation effect. These results provide novel insight into the conditions under which NA translates into delusional beliefs. The finding that emotion awareness and rumination have a relevant role corresponds with current psychological conceptualisations of psychosis and with the at‐ tempt to treat delusions by focusing on reducing ruminative thoughts. To investigate the causal effect, treatment trials with a focus on enhancing these components of emotion regulation are needed.
Ludwig, Mehl, Schlier, et al. (2020)	Germany Cross‐sectional and Short‐Term Longitudinal Study EMA (6 days)	71 PD (57.8% M/42.2% F) 42 HCs (Gender unclear) Outpatient	PD 37.80 (12.15) HCs 37.14 (13.56)	Ecological Momentary Assessment (EMA) Positive and Negative Syndrome Scale (PANSS) Calgary Rating Scale for SZ (CDSS) Role Functioning Scale (RFS) Emotion Regulation Questionnaire (ERQ) Emotion‐Regulation Skills Questionnaire (ERSQ‐27)	Questionnaires of habitual ER were largely predictive of affect in daily life. There was indication of a more frequent use of putatively maladaptive strategies but either no differences in individual adaptive strategies or even a more frequent use (reappraisal) in PDs compared to NCs. Several ER strategies (e.g., reappraisal and rumination) proved effective in reducing NA by the next prompt, independent of group, but suppression was effective in only PDs and acceptance had unfavourable effects in both groups. Thus, PDs demonstrated an increased use of ER strategies in daily life, of which the majority helped them to reduce NA. These findings indicates that their increased levels of NA are not explicable by difficulties in deploying explicit ER strategies.
Lysaker et al. (2011)	USA Cross‐sectional	101 SZ (86 M/15 F) Outpatient and Inpatient	46.26 (9.66)	Indiana Psychiatric Illness Interview (IIPI) Metacognition Assessment Scale (MAS) Bell–Lysaker Emotional Recognition Task (BLERT) Positive and Negative Syndrome Scale (PANSS) Wisconsin Card Sorting Test (WCST) Wechsler Adult Intelligence Scale III (WAIS‐III) Hopkins Verbal Learning Test (HVLT) Conners Continuous Performance Test II (CPT‐II) Trauma Assessment for Adults—Brief Revised Version (TAA)	Results indicated that the group unaware of their own emotions and those of others demonstrated poorer verbal memory, processing speed, executive function, less emotional discomfort and higher levels of disorganisation symptoms relative to the other groups. The group aware of their own emotions but not those of others had a significantly higher report of childhood sexual abuse.
Masillo et al. (2012)	UK Cross‐sectional	62 ARMS (37 M/25 F) 39 HCs (20 M/19 F) Outpatient	ARMS 22.63 (4.05) HCs 24.03 (4.22)	Interpersonal Sensitivity Measure (IPSM) Prodromal Questionnaire (PQ) Ways of Coping Questionnaire (WCQ) Depression Anxiety Stress Scales (DASS)	Individuals with an ARMS reported higher interpersonal sensitivity compared to HCs. Associations between interpersonal sensitivity, positive psychotic symptoms (i.e., paranoid ideation), avoidant coping and symptoms of depression, anxiety and stress were also found. These findings suggest that being ‘hypersensitive’ to interpersonal interactions is a psychological feature of the putatively prodromal phase of psychosis. The relationship between interpersonal sensitivity, attenuated positive psychotic symptoms, avoidant coping and negative emotional states may contribute to long‐term deficits in social functioning. We illustrate the importance, when assessing a young client with a possible ARMS, of examining more subtle and subjective symptoms in addition to attenuated positive symptoms.
Meyer (2001)	USA Longitudinal (4–6 weeks follow up)	39 SZ 31 Clinical but non‐SZ (46 M/24 F) Inpatient	SZ 39.46 (10.29) Clinical but non‐SZ 42.26 (13.82)	The Brief Cope Brief Psychiatric Rating Scale (BPRS) Modified Hamilton Rating Scale for Depression (MHRSD) Bech–Rafaelsen Mania Rating Scale (BRMS) UCLA Social Attainment Survey (SAS) Psychological Well‐Being Questionnaire (PWBQ)	Among patients with SZ, psychosis symptom severity correlated inversely with adaptive coping (e.g., acceptance, planning and seeking support) but did not correlate with maladaptive coping (e.g., self‐blame and denial). Among those with SZ, deficits in adaptive coping also predicted relative increases in psychosis symptoms over time, controlling for intake symptom severity. These findings suggest that among patients without SZ, maladaptive coping correlated concurrently with depressive symptoms. Several hypothesized associations between concurrent coping, functioning, and well‐being were also documented.
Moran et al. (2018)	USA Cross‐sectional	30 SZ (52% M/48% F) Outpatient	39.48 (8.40)	Psychosis Subscale of the Brief Psychiatric Rating Scale (BPRS Psychosis) Expression Subscale of the Clinical Assessment Interview for Negative Symptoms (CAINS EXP) Motivation and Pleasure Subscale of the Clinical Assessment Interview for Negative Symptoms (CAINS MAP) Emotion Regulation Questionnaire (ERQ) The Wechsler Abbreviated Scale of Intelligence (WASI) Savouring Beliefs Inventory (SBI) Specific level of functioning Interpersonal Relationship Sub‐Scale (SLOF) Social interaction = mean number of people interacted with daily Social interest = mean interest in daily social interaction EMA rating positive and NA	Hierarchical linear modelling revealed that self‐reported use of cognitive reappraisal and savouring of emotional experiences were related to greater positive emotion in daily life. In contrast, self‐reported suppression was related to greater negative emotion, reduced positive emotion, and reduced social interaction in daily life. These findings suggest that individual differences in habitual emotion regulation strategy usage have important relationships to everyday emotional and social experiences in SZ.
Moritz et al. (2016)	Germany Cross‐sectional	1100 HCs (32% M/68% F) 100 Depression (23% M/77% F) 75 SZ (36% M/64% F) Inpatient	HCs 41.96 (11.35) Depression 42.59 (10.47) SZ 40.89 (9.41)	Maladaptive and Adaptive Coping Style Questionnaire (MAX) Patient Health Questionnaire (PHQ‐9) Community Assessment of Psychic Experiences Scale (CAPE)	SZ patients showed compromised coping abilities relative to HCs, particularly a lack of engaging in adaptive coping. Depression was more closely tied to dysfunctional coping than were positive symptoms as indicated by group comparisons and correlational analyses. Correlations between positive symptoms, particularly paranoid symptoms, and avoidance and suppression remained significant when depression was controlled for. These findings suggest that although maladaptive and adaptive coping are unlikely to represent proximal mechanisms for the pathogenesis of positive symptoms, fostering coping skills may reduce positive symptoms via the improvement of depressive symptoms, which are increasingly regarded as risk factors for core psychotic symptoms. Further‐ more, the reduction of avoidance and suppression may directly improve positive symptoms.
Myin‐Germeys et al. (2001)	Netherlands Short‐Term Longitudinal Study (6 days)	42 psychosis (22 M/20 F) 47 first‐degree relatives (25 M/22 F) 49 HCs (24 M/25 F) Inpatient and Outpatient	Psychosis 31.9 (7.7) First‐degree relatives 36.5 (10.7) HCs 35.2 (8.9)	Ecological Momentary Assessment (EMA)	Multilevel regression analyses showed that an increase in subjective stress was associated with an increase in NA and a decrease in PA in all groups. However, the groups differed quantitatively in their pattern of reactions to stress. Patients with psychotic illness reacted with more intense emotions to subjective appraisals of stress in daily life than HCs. The decrease in PA in the relatives was similar to that of the patients, while the increase in NA in this group was intermediary to that of patients and HCs. Higher levels of familial risk for psychosis were associated with higher levels of emotional reactivity to daily life stress in a dose–response fashion. Subtle alterations in the way persons interact with their environment may constitute part of the vulnerability for psychotic illness.
Myin‐Germeys et al. (2003)	Netherlands Short‐Term Longitudinal Study (6 days)	42 Non‐affective psychosis (NAP; 22 M/20 F) 45 Bipolar Disorder (BPD; 19 M/19 F) 46 Major Depressive Disorder (MDD; 20 M/26 F) 49 HCs (24 M/25 F) Outpatient	NAP 31.9 (No SD) BPD 46.2 (No SD) MDD 40.3 (No SD) HCs 35.2 (No SD)	Ecological Momentary Assessment (EMA) Hamilton Rating Scale for Depression (HRSD) Brief Psychiatric Rating Scale (BPRS)	Multilevel regression analyses showed an increase in NA in MDD, a decrease in PA in BD and both an increase in NA and a decrease in PA in NAP in association with the subjectively stressful situations, compared with HCs. Individuals with NAP, MDD and BD display differences in emotional stress reactivity. Type of mood disorder may exert a pathoplastic effect on emotional reactivity in individuals with MDD and BD. Individuals with NAP may be most vulnerable to the effects of daily life stress.
Myin‐Germeys et al. (2005)	Netherlands Cross‐sectional Short‐Term Longitudinal Study (6 days)	42 PD (in clinical remission) (22 M/20 F) 47 First‐degree relatives (25 M/22 F) 49 HCs (24 M/25 F) Inpatient and Outpatient	PD 31.9 (7.7) First‐degree relatives 36.5 (10.7) HCs 35.2 (8.9)	Ecological Momentary Assessment (EMA)	Multilevel regression analyses revealed significant increases in psychosis intensity associated with increases in subjective activity—and event‐related stress in patients. First‐degree relatives reported increases in psychosis intensity in relation to activity‐related stress but not event‐ related stress. No association was found in HCs. These findings suggest that individuals at increased risk for psychosis show continuous variation in the intensity of subtle psychotic experiences associated with minor stresses in the flow of daily life. Behavioural sensitization to environmental stress may therefore be a vulnerability marker for SZ, reflecting dopaminergic hyper‐responsivity in response to environmental stimuli.
Nittel et al. (2018)	Germany Cross‐sectional Short‐Term Longitudinal Study (6 days)	32 Psychosis (14 M/18 F) Outpatient	Psychosis 35.87 (11.05)	Ecological Momentary Assessment (EMA) Positive and Negative Syndrome Scale (PANSS) Paranoia Checklist (PCL) German Mehrfachwahl Wortschatz Test Version B (MWT‐B)	Hierarchical linear regression analysis revealed that patients with psychosis who presented pronounced instability of negative emotions showed more severe levels of state paranoia. In addition, patients with psychosis who used expressive suppression when confronted with negative emotions at one point in time presented more pronounced levels of state paranoia at the following point in time. The results presented here suggest that both emotional instability and the use of expressive suppression might cause state paranoia and thus add to our understanding of causal mechanisms related to paranoia such as instability of negative emotions and the use of less adaptive ER strategies.
Nowak et al. (2022)	Germany Short‐Term Longitudinal Study (7 days)	32 PD (50% M/50% F) 37 Attenuated psychotic symptoms (AS; 49% M/51% F) 35 Obsessive Compulsive Disorder (OCD; 44% M/66% F) HCs (51% M/49% F) Setting not specified	PD 38.03 (8.74) AS 31.08 (10.79) OCD 36.66 (11.27) HCs 37.31 (11.03)	Ecological Momentary Assessment (EMA) Structured Clinical Interview for DSM‐IV (SCID) Positive and Negative Syndrome Scale (PANSS) Community Assessment of Psychic Experiences Scale (CAPE) Obsessive Compulsive Inventory‐Revised (OCI‐R)	Mixed‐model ANOVAs revealed higher instability in both PSY and OCD compared to HCs, but no significant differences for variability and inertia. AS had an intermediate position and did not differ significantly from any other group. We found evidence for small to medium effect sizes for the influence of mean affect levels on the dynamic indicators. These findings indicate that individuals with psychotic disorders have increased affective instability and that this could be a transdiagnostic phenomenon. Zooming in on the variability and inertia components did not confer additional benefits. Emotion‐focused interventions for psychosis should focus on reducing frequent and extreme affective fluctuations.
Orth et al. (2022)	USA Cross‐sectional Short‐Term Longitudinal Study (7 days)	32 SZ (18 M/14 F) Outpatient	SZ 41.66 (12.94)	Ecological Momentary Assessment (EMA) Expanded Brief Psychiatric Rating Scale (BPRS) The Green Paranoid Thoughts Scales (GPTS)	Multilevel modelling results indicated that greater NA is associated with higher concurrent levels of paranoid ideation and that it is marginally related to elevated levels of future paranoid ideation. In contrast, PA was unrelated to momentary experiences of paranoid ideation. More severe momentary paranoid ideation was also associated with an elevated desire to withdraw from social encounters, irrespective of when with familiar or unfamiliar others. These observations underscore the role of NA in promoting paranoid ideation and highlight the contribution of paranoid ideation to the motivation to socially withdraw in psychotic disorders.
Painter et al. (2019)	USA Cross‐sectional	25 SZ (15 M/10 F) 21 HCs (12 M/9 F) Setting not specified	SZ 48.04 (11.88) HCs 51.71 (8.34)	Film stimuli Facial Expression Coding System (FACES) Differential Emotions Scale (DES)	Although people with SZ showed increased positive expressivity following amplification and decreased negative emotion experience following reappraisal, overall, they expressed less positive emotion and experienced more negative emotion compared with HCs. Neither group was effective at physiological suppression. Together these findings suggest that people with SZ can engage in amplification and reappraisal when explicitly instructed to do so, albeit additional practice may be necessary to modify emotion responses to levels similar to HCs.
Palmier‐Claus et al. (2012)	UK Cross‐sectional Short‐Term Longitudinal Study (6 days)	27 Psychosis (21 M/6 F) 27 UHR (14 M/13 F) 27 HCs (14 M/13 F) Inpatient and Outpatient	Psychosis 33.2 (11.0) UHR 22.6 (4.4) HCs 22.6 (5.2)	Ecological Momentary Assessment (EMA) Perceived Stress Scale (PSS)	Multilevel regression analyses showed that individuals at UHR of developing psychosis reported greater negative emotions in response to stress than the HCs. UHR individuals also experienced greater emotional reactivity to stress when compared with the psychosis group. No significant differences were observed between the psychosis group and HCs. Stress measures significantly predicted the intensity of psychotic symptoms in UHR individuals and the psychosis group, but the extent of this did not significantly differ between the groups. Individuals at UHR of developing psychosis may be particularly sensitive to everyday stressors. This effect may diminish after transition to psychosis is made and in periods of stability. Subtle increases in psychotic phenomena occur in response to stressful events across the continuum of psychosis.
Parrish et al. (2023)	USA Short‐Term Longitudinal Study (7 days)	105 SZ (71.2% M/28.8% F) Outpatient	51.92 (9.2)	Ecological Momentary Assessment (EMA)	Forty five percent of surveys reported the absence of NA, though there was an inverse within‐subjects correlation between PA and NA. Between‐subjects, there was a large inverse correlation between PA and NA. Greater mood variability was associated with a greater number of social interactions. The results of this study point to both the role of social context in mood variability, and momentary trends in mood experiences, with some individuals reporting no NA, some indicating both PA and NA, and some indicating a more normative affect pattern. Later research should address the possible impact of emotion perception bias and social interactions on moods states in SZ.
Perry et al. (2011)	Sydney Cross‐sectional	33 SZ 36 HCs (Gender unclear) Outpatient	SZ 43.7 (9.89) HCs 40.8 (11.49)	Wechsler Abbreviated Scale of Intelligence (WASI) Scale for the Assessment of Negative Symptoms (SANS) Scale for the Assessment of Positive Symptoms (SAPS) Social Functioning Scale Depression Anxiety Stress Scales (DASS) Emotion Regulation Questionnaire (ERQ) Acceptance and action questionnaire (AAQ)	No group differences were found in terms of use of suppression or reappraisal, but clinical participants reported using less acceptance. Further, greater use of acceptance was associated with better psychosocial outcomes. These results highlight the potential utility of acceptance‐based interventions for comorbid psychopathology in SZ.
Piotrowski et al. (2019)	Poland Cross‐sectional	42 FEP (21 M/21 F) 28 SZ‐AR (14 M/14 F) 37 FHR‐P (12 M/25 F) 40 HCs (15 M/25 F) Outpatient	FEP 27.7 (7.3) SZ‐AR 43.0 (13.5) FHR‐P 36.9 (11.0) HCs 27.8 (8.5)	Positive and Negative Syndrome Scale (PANSS) Montgomery‐Asberg Depression Rating Scale (MADRS) Young Mania Rating Scale Global Assessment of Functioning (GAF) Social and Occupational Functioning Assessment Scale (SOFAS) Coping Inventory for Stressful Situations (CISS) Perceived Stress Scale (PSS) The list of threatening experiences (LTE)	Individuals with FEP were less likely to use task‐focused coping, while SCZ‐AR subjects preferred using distraction when compared to HCs. Both groups of participants did not differ significantly in terms of using specific coping styles. No significant differences in the use of various coping strategies between FHR‐P individuals and HCs were found. Higher odds of using emotion‐focused coping and distraction were associated with more severe depressive symptoms in individuals with psychosis. Moreover, higher frequency of using distraction was associated with worse functioning in individuals with psychosis. However, this association appeared to be insignificant after adjustment for multiple testing. These findings highlight that coping styles are similar in FEP and SCZ‐AR subjects. However, decreased use of task‐focused coping is more specific for FEP individuals while a preference of distraction might be more typical for SCZ‐AR individuals. The use of various coping styles is similar in FHR‐P individuals and HCs. Preference of distraction and emotion‐focused coping might be related to more severe depressive symptoms and poor functioning in individuals with psychosis.
Raugh and Strauss (2022)	USA Short‐Term Longitudinal Study (6 days)	48 SZ (17 M/31 F) 52 HCs (12 M/36 F) Outpatient	SZ 38.56 (11.86) HCs 38.94 (10.26)	Ecological Momentary Assessment (EMA)	Results indicated that SZ identified the need to regulate at a higher rate than HCs. Specifically, SZ displayed an inefficient threshold for identifying the need to regulate, such that they regulated too much when NA was low and too little when NA was high. Emotion regulation effort exertion was also inefficient, such that effort was too high at low levels of NA and too low at high levels of NA in SZ. These identification stage abnormalities also demonstrated differential associations with positive and negative symptoms. Findings suggest that identification stage abnormalities may create a bottleneck that feeds forward and impacts subsequent stages of emotion regulation in SZ that are critically related to symptoms. Targeting the psychological processes underlying these identification stage abnormalities might offer a novel means of treating positive and negative symptoms in SZ.
Raugh et al. (2025)	USA Short‐Term Longitudinal Study (6 days)	39 SZ (13 M/26 F) 34 HCs (9 M/25 F) Outpatient	SZ 38.85 (10.3) HCs 39.06 (10.95)	Ecological Momentary Assessment (EMA)	PA down‐regulation rates were similarly low in both groups. The intensity of PA did not interact with group for identification rate, strategies selected, or implementation effectiveness, suggesting that PA up‐regulation was relatively normal in SZ. However, the intensity of NA predicted the need to up‐regulate PA, suggesting that regulation of PA was initiated in response to NA. Further, this relationship differed between the groups such that SZ regulated PA more frequently and with more effort at lower levels of NA. Yet, these regulatory attempts were ineffective at decreasing the severity of anhedonia, and SZ were less effective than HCs at decreasing the intensity of NA. Overall, the pattern of emotion regulation abnormalities observed in SZ differs based upon regulatory goals. Abnormalities in regulating NA may be more central to psychopathology in SZ than abnormalities in PA.
Reininghaus et al. (2016)	UK Short‐Term Longitudinal Study (6 days)	51 FEP (28 M/23 F) 46 ARMS (21 M/25 F) 53 HCs (25 M/28 F) Setting not specified	FEP 28.3 (8.6) ARMS 23.6 (4.7) HCs 35.0 (12.6)	Ecological Momentary Assessment (EMA)	In all three groups, elevated stress sensitivity, aberrant salience, and enhanced threat anticipation were associated with an increased intensity of psychotic experiences. However, elevated sensitivity to minor stressful events, activities, and areas and enhanced threat anticipation were associated with more intense psychotic experiences in FEP individuals than HCs. Sensitivity to outsider status and aberrantly salient experiences were more strongly associated with psychotic experiences in ARMS individuals than HCs. These findings suggest that stress sensitivity, aberrant salience, and threat anticipation are important psychological processes in the development of psychotic experiences in daily life in the early stages of the disorder.
Ritsner et al. (2006)	Israel Cross‐sectional	237 SZ (188 M/49 F) 175 HCs (65 M/110 F) Inpatient	SZ 37.9 (9.9) HCs 38.4 (10.0)	The Coping Inventory for Stressful Situations (CISS) The Positive and Negative Syndrome Scale (PANSS) The Talbieh Brief Distress Inventory (TBDI) The Quality of Life Enjoyment and Satisfaction Questionnaire (Q‐LES‐Q) The General Self‐Efficacy Scale (GSES) The Rosenberg Self‐Esteem scale (RSES) Multidimensional Scale of Perceived Social Support (MSPSS)	Emotion‐oriented coping style and emotional distress were significantly higher in the SZ group, whereas the task‐oriented coping style, self‐efficacy, perceived social support and satisfaction with quality of life were lower compared with HCs. When eight CISS coping patterns were defined, the results revealed that patients used emotion coping patterns 5.5 times more frequently, and task and task‐avoidance coping patterns significantly less often than HCs. Coping patterns have different associations with current levels of dysphoric mood and emotional distress, self‐construct variables, and satisfaction with quality of life. Thus, the identified coping patterns may be an additional useful presentation of the diversity of coping strategies used by SZ patients. Coping patterns may be considered an important source of knowledge for patients who struggle with the illness and for mental health professionals who work with SZ patients.
Rudnick (2001)	Israel Cross‐sectional	58 SZ (40 M/18 F) Outpatient	42.4 (10.8)	Positive and Negative Syndrome Scale (PANNS) Ways of Coping Checklist Wisconsin Quality of Life Index (W‐QLI)	Negative symptoms were inversely related to activities of daily living, and positive symptoms were directly related to distress. There were no other significant relations between symptoms and quality of life. Problem‐focused and emotion‐focused coping did not moderate the relation between symptoms and quality of life. Further study is required concerning coping in SZ.
Ryan et al. (2021)	Republic of Ireland Longitudinal 8 Sessions over 4 weeks (One month follow up)	55 PD (33 M/22 F) Inpatient and Outpatient	36.04 (12.78)	The Group Climate Scale—Short form The Difficulties with Emotional Regulation Scale (DERS) The Southampton Mindfulness Questionnaire (SMQ) The Psychotic Symptom Rating Scales (PSYRATS) The Fear of Recurrence Scale (FORSE) The Recovery Assessment Scale (RAS)	High group attendance and cohesion support the acceptability of the group. Participants reported less difficulty with Emotional Regulation, increased mindful relating to distressing symptoms, and improvements in recovery dimensions from pre‐to post‐intervention, and maintained at one‐month follow‐up. Participants' hallucinations and delusions reduced from pre‐intervention to follow‐up. There was no change in fear of relapse.
Smith et al. (2006)	UK Cross‐sectional	100 SZ (68 M/32 F) Outpatient	39 (10.9)	Scale for the Assessment of Positive Symptoms (SAPS) Positive and Negative Syndrome Scale (PANSS) Psychotic Symptom Rating Scales (PSYRATS) Beck Depression Inventory‐II (BDI‐II) Rosenberg Self‐esteem Scale (RSES) Brief Core Schema Scales (BCSS)	Analysis indicated that individuals with more depression and lower self‐esteem had auditory hallucinations of greater severity and more intensely negative content and were more distressed by them. In addition, individuals with more depression, lower self‐esteem and more negative evaluations about themselves and others had persecutory delusions of greater severity and were more pre‐occupied and distressed by them. The severity of grandiose delusions was related inversely to depression scores and negative evaluations about self, and directly to higher self‐esteem. This study provides evidence for the role of emotion in SZ spectrum‐disorders. Mood, self‐esteem and negative evaluative beliefs should be considered when conceptualising psychosis and designing interventions.
Strakeljahn et al. (2023)	Short‐Term Longitudinal Study (14 days)	43 Attenuated psychosis symptoms (37.21% M/62.79% F) 40 HCs (35% M/65% F) Setting not specified	Attenuated psychosis symptoms 21.49 (2.24) HCs 21.32 (1.59)	Ecological Momentary Assessment (EMA) Emotion‐Regulation Skills Questionnaire (ERSQ‐ES) Community Assessment of Psychic Experiences (CAPE)	Individuals with attenuated psychosis symptoms used multiple tolerance‐based adaptive ER‐strategies (acceptance, understanding, clarity, directing attention) less frequently in daily life. However, only a single change‐focused adaptive ER‐strategy (modification) showed consistently lower utilisation rates in individuals with attenuated psychosis symptoms This indicates that people with an elevated risk of psychosis use various adaptive ER‐strategies focusing on comprehending and accepting negative emotions less frequently. Fostering these strategies with targeted interventions could promote resilience against transitioning into psychosis.
Strauss et al. (2019)	USA Short‐Term Longitudinal Study (6 days)	30 SZ (gender not specified) Outpatient	Age not specified	Ecological Momentary Assessment (EMA)	Markov chain analysis indicated that although SZ tried to implement emotion regulation strategies frequently during psychotic experiences, those attempts were ineffective at reducing negative emotion from one time point to the next. Network analysis indicated that patients who were less effective at regulating their emotions during psychotic experiences had more dense connections among individual emotions. Findings indicate that psychotic experiences are associated with abnormally strong connections among discrete emotional states that are difficult to regulate despite efforts to do so.
Strauss et al. (2019)	USA Cross‐sectional	Study 1: 262 PLEs (51.15% M/48.85% F) 1226 HCs (51.63% M/48.37% F) Study 2: 29 CHR (31.03% M/68.97% F) 29 HCs (20.70% M/79.30% F) Study 3: 61 SZ (63.90% M/36.10% F) 67 HCs (69.12% M/30.88% F) Outpatient	Study 1: PLEs 14.25 (2.22) HCs 14.14 (2.04) Study 2: CHR 19.24 (2.49) HCs 19.52 (1.24) Study 3: SZ 39.98 (12.18) HCs 40.57 (11.96)	Emotion Regulation Questionnaire for Children and Adolescents (ERQ‐CA) Youth Psychosis At‐Risk Questionnaire‐Brief (YPARQ‐B) Strengths and Difficulties Questionnaire (SDQ)	The three psychosis groups did not differ from each other in reported use of suppression; however, there was evidence for a vulnerability‐related dose‐dependent decrease in reappraisal. Across each sample, lower use of reappraisal was associated with poorer clinical outcomes. Findings indicate that emotion regulation abnormalities occur across a continuum of psychosis vulnerability and represent important targets for intervention.
Strauss et al. (2023)	USA Short‐Term Longitudinal Study (6 days)	46 SZ (34.8% M/65.2% F) 52 HCs (30.8% M/69.2% F) Outpatient	SZ 38.9 (10.3) HCs 38.5 (12.0)	Ecological Momentary Assessment (EMA) Brief Negative Symptom Scale (BNSS) Positive and Negative Syndrome Scale (PANSS) Level of Functioning Scale Young Mania Rating Scale (YMRS)	Results indicated that less dense emotion networks were associated with greater severity of negative symptoms, whereas more dense emotion networks were associated with more severe positive symptoms and mania. Additionally, SZ evidenced greater centrality for shame, which was associated with greater severity of positive symptoms. These findings suggest that positive and negative symptoms are associated with distinct profiles of temporally dynamic and interactive emotion networks in SZ. Findings have implications for adapting psychosocial therapies to target specific discrete emotional states in the treatment of positive versus negative symptoms.
Tarrier et al. (2007)	UK Cross‐sectional	278 SZ (193 M/85 F) Inpatient and Outpatient	29.6 (10.2)	The Health of the Nation Outcome Scales (HoNOS) Positive and Negative Syndrome Scale (PANSS)	Emotional withdrawal, but not blunted affect was significantly and negatively associated, and depression positively associated with suicide behaviour. There was evidence to indicate that restricted emotions are associated with reduced suicide risk as predicted.
Thewissen et al. (2011)	Netherlands Short‐Term Longitudinal Study (6 days)	33 Paranoid patients (26 M/7 F) 34 Non‐Paranoid patients (26 M/8 F) 15 Remitted patients (14 M/1 F) 39 High schizotypy patients (14 M/25 F) 37 HCs (14 M/23 F) Setting not specified	Paranoid patients 38.7 (10.5) Non‐Paranoid patients 36.0 (11.6) Remitted patients 32.5 (12.3) High schizotypy patients 47.4 (10.2) HCs 48.7 (9.2)	Ecological Momentary Assessment (EMA) Positive and Negative Syndromes Scale (PANNS) Paranoia Scale (PS)	Specific aspects of emotional experience were implicated in the onset and persistence of paranoid episodes. Both an increase in anxiety and a decrease in self‐esteem predicted the onset of paranoid episodes. Cross‐sectionally, paranoid episodes were associated with high levels of all negative emotions and low level of self‐esteem. Initial intensity of paranoia and depression was associated with longer, and anger/irritability with shorter duration of paranoid episodes. Paranoid delusionality is driven by negative emotions and reductions in self‐esteem, rather than serving an immediate defensive function against these emotions and low self‐esteem. Clinicians need to be aware of the central role of emotion‐related processes and especially self‐esteem in paranoid thinking.
van der Steen et al. (2017)	Belgium Netherlands Short‐Term Longitudinal Study (6 days)	24 non‐affective psychotic disorder (15 M/9 F) 22 CHR (17 M/5 F) 26 HCs (16 M/100 F) Outpatient	Non‐affective psychotic disorder 33.9 (8.8) CHR 25.2 (2.0) HCs 24.5 (3.6)	Ecological Momentary Assessment (EMA)	Multilevel models showed significantly larger associations between NA and activity‐related stress for CHR patients than for psychotic patients (*p* = 0.008) and for CHR compared to HCs (*p* < 0.001). Similarly, the association between activity‐related stress and psychotic symptoms was larger in CHR than in psychotic patients (*p* = 0.02). Finally, the association between NA and symptoms (*p* < 0.001) was larger in CHR than in psychotic patients. Stress sensitization seems to play a role particularly in the early phase of psychosis development as results suggest that CHR patients are more sensitive to daily life stressors than psychotic patients. In this early phase, psychotic experiences also contributed to the experience of stress.
Vines et al. (2021)	USA Cross‐sectional	32 CHR (17 M/15 F) 42 HCs (21 M/21 F) 13 SZ (5 M/8 F) Setting not specified	CHR 18.5 (2.9) HCs 17.8 (2.8) SZ 17.2 (1.7)	Structured Interview for Psychosis—Risk Syndromes (SIPS) Global Functioning: Social Scale (GFS) Global Functioning: Role Scale (GFR) The Emotion Regulation Questionnaire (ERQ) Difficulty in Emotion Regulation Scale (DERS) Wechsler Abbreviated Scale of Intelligence (WASI) Emotion Reactivity Scale (ERS)	CHR and AOP endorsed experiencing heightened levels of emotion reactivity and greater difficulty utilising emotion regulation strategies compared to TD. These impairments were stable across time and adolescent development. Greater levels of emotion reactivity were associated with greater emotion regulation impairments. Greater impairments in emotion regulation were associated with lower social functioning and greater negative symptom severity. Therapeutic interventions designed to reduce emotion reactivity and improve one's ability to utilise emotion regulation strategies may be effective in reducing clinical symptomatology and improving real‐world functioning in CHR and AOP.
Visser et al. (2018)	USA Short‐Term Longitudinal Study (6 days)	28 SZ (57.1% M/42.9% F) 28 HCs (64.3% M/35.7% F) Outpatient	SZ 41.39 (10.76) HCs 43.75 (11.75)	Ecological Momentary Assessment (EMA) Study Brief Negative Symptom Scale (BNSS) Brief Psychiatric Rating Scale (BPRS) Ecological Momentary Assessment (EMA) Psychotic Symptoms Rating Scale (PSYRATS) Level of Function Scale (LOF)	Results indicated that SZ demonstrated adequate emotion regulation effort, but poor effectiveness. Abnormalities were observed at each of the three stages of the emotion regulation process. At the identification stage, SZ initiated emotion regulation efforts at a lower threshold of negative emotion intensity. At the selection stage, SZ selected more strategies than HCs and strategies attempted were less contextually appropriate. At the implementation stage, moderate to high levels of effort were ineffective at decreasing negative emotion. These findings suggest that although SZ attempt to control their emotions using various strategies, often applying more effort than HCs, these efforts are unsuccessful; emotion regulation abnormalities may result from difficulties at the identification, selection, and implementation stages.
Vodušek et al. (2014)	Denmark Qualitative (6 months)	20 FEP (60% M/40% F) Setting not specified	22.1 (4.9)	Phenomenological hermeneutics	The emotion experiences described vary greatly in both quality and intensity, but appear to have a common phenomenology. Anxiety is reported as the basic emotion which buffers, transforms and sometimes supplants all others. Emotions in general are experienced as foreign, unstable and perturbing, thereby contributing greatly to feelings of ambivalence, perplexity and an unstable sense of self in general. The findings of this study have important therapeutic and theoretical implications because they suggest that emotion experiences in SZ spectrum disorders may underlie a wide range of psychopathological phenomena in both the cognitive and social functioning domains. Due to the relatively small sample size and its selection from psychotherapeutic units, the results may not be generalizable to all SZ patients.
Yan Lam et al. (2020)	Hong Kong Cross‐sectional	Total sample: 46 SZ (11 M/35 F) 24 Intervention Group (6 M/18 F) 22 HCs (5 M/17 F)	Total sample 25–34 = 4 35–44 = 5 45–54 = 20 55+ = 17 Intervention Group: 25–34 = 1 35–44 = 2 45–54 = 10 55+ = 11 HCs: 25–34 = 3 35–44 = 3 45–54 = 10 55+ = 6 (No mean or SD stated)	The mindfulness‐based psychoeducation programme (MBPP) Emotion regulation questionnaire (ERQ) Short Ruminative Response Scale (SRRS) Depression Anxiety Stress Scale (DASS‐21) Chinese version of Psychotic Symptom Rating Scale (C‐PSYRATS) Five Facet Mindfulness Questionnaire—Short form (FFMQ‐SF) Self‐Assessment of Negative Symptoms (SANS)	The results of the Generalised Estimating Equations test indicated that the Mindfulness Based Psychoeducation Programme (MBPP) group showed a significant improvement in reappraisal at a three‐month follow‐up, and a significant reduction in rumination across time. However, the Generalised Estimating Equations indicated no significant difference in rumination and expressive suppression in the MBPP group. Two participants reported having unwanted experiences, including feelings of terror and distress during the mindfulness practice. The MBPP appeared to be effective for improving emotion regulation, which will contribute to future large‐scale RCT to confirm the treatment effects in more diverse groups of SZ patients.
Yee et al. (2019)	USA Cross‐sectional	Sample 1: 56 SZ (30 M/26 F) 34 HCs (13 M/21 F) Sample 2: 50 CHR (29 M/21 F) 56 HCs (22 M/34 F) Outpatient	Sample 1: SZ 39.30 (9.72) HCs 37.79 (12.43) Sample 2: 50 CHR 18.94 (1.82) 56 HCs 19.16 (2.62)	Scale for the Assessment of Positive Symptoms (SAPS) Scale for the Assessment of Negative Symptoms (SANS) Differential Emotions Scale IV‐A (DES) Brief Psychiatric Rating Scale (BPRS) Global Assessment of Functioning (GAF) Beck Depression Inventory (BDI) Beck Anxiety Inventory (BAI) Global Functioning Scale: Social (GFS‐S) and Role (GFS‐R) Modified Differential Emotions Scale (mDES)	Both clinical groups reported lower levels of PA (specific to joy among individuals with SZ) and higher levels of NA compared with HCs. For individuals with SZ, links were found between PA and negative symptoms (which remained after controlling for secondary factors) and between NA and positive symptoms. For individuals at CHR, links were found between both affect dimensions and both types of symptoms (which were largely accounted for by secondary factors). Both clinical groups showed some evidence of reduced trait PA and elevated trait NA, suggesting that increasing trait PA and reducing trait NA is an important treatment goal across both populations. Clinical correlates of these emotional abnormalities were more integrally linked to clinical symptoms in individuals with SZ and more closely linked to secondary influences such as depression and anxiety in individuals at CHR.
Zappia et al. (2012)	Italy Cross‐sectional	47 SZ (23 M/24 F) Outpatient	43.0 (11.6)	Positive and Negative Syndrome Scale (PANSS) Calgary Depression Scale for SZ (CDSS) Scale for the Assessment of Unawareness of Mental Disorder (SUMD) Rosenberg Self‐Esteem Scale (RSES) Quality of Life Scale (QLS) Short Form Health Survey 36 (SF‐36) questionnaire Coping Inventory for Stressful Situations (CISS)	From the multiple regression analysis, depressive symptoms and objective quality of life were found to be contributing factors to the task‐oriented coping style, with an explained variance of approximately 32%. Negative symptomatology, subjective quality of life, self‐esteem, awareness of psychosis symptoms and their attribution to the illness were found to be contributing factors to emotion‐oriented coping strategies, with an explained variance of approximately 60%. These results revealed the role of some clinical and functional variables as contributing factors to individual coping styles. From this perspective, the use of support and rehabilitation interventions and cognitive‐behavioural therapies, aimed at managing psychotic symptoms and stressful events, could direct patients to adopt more adaptive strategies, positively influencing outcome indices.
Zarbo, Rota, et al. (2023)	Italy Short‐Term Longitudinal Study (7 days)	53 SZ Inpatient (37 M/16 F) 46 SZ Outpatient (27 M/19 F) 111 HCs (67 M/44 F) Inpatient and Outpatient	SZ Inpatient 43.4 (9.9) SZ Outpatient 38.1 (10.4) HCs 41.8 (10.0)	Ecological Momentary Assessment (EMA) Brief Psychiatric Rating Scale (BPRS) Brief Negative Symptom Scale (BNSS) Specific Levels of Functioning Scale (SLOF)	Patients with SSD, especially those living in residential facilities, spent more time being sedentary, and self‐reported more sedentary and self‐care activities, experiencing higher levels of negative emotions compared with HCs. Moreover, higher functioning levels among patients were associated with more time spent in moderate‐to‐vigorous activity. Sedentary behaviour and negative emotions are particularly critical among patients with SSD and are associated with more impaired clinical outcomes. Mobile‐EMA and wearable sensors are useful for monitoring the daily life of patients with SSD and the level of PA. This population needs to be targeted with specific rehabilitative programmes aimed at improving their commitment to structured daily activities.
Zarbo, Zamparini, et al. (2023)	Italy Short‐Term Longitudinal Study (7 days)	57 SZ Inpatient (40 M/17 F) 46 SZ Outpatient (27 M/19 F) 112 HCs (68 M/44 F) Inpatient and Outpatient	SZ Inpatient 43.4 (9.9) SZ Outpatient 38.1 (10.4) HCs 41.8 (10.0)	Ecological Momentary Assessment (EMA) Brief Psychiatric Rating Scale (BPRS) Brief Negative Symptom Scale (BNSS) Specific Levels of Functioning Scale (SLOF)	Participants with SSD, and especially residential patients, had a higher intensity of negative emotions when compared to HCs. Moreover, all people with SSD reported a greater between‐person‐variability of both positive and negative emotions and greater intra‐variability of negative emotions than HCs. In addition, the emotion variability in people with SSD does not follow a linear or quadratic trend but is more ‘chaotic’ if compared to HCs. Adequate assessments of positive and negative emotional experiences and their time course in people with SSD can assist mental health professionals with well‐being assessment, implementing targeted interventions through the identification of patterns, triggers, and potential predictors of emotional states.

Abbreviations: ACS, Adaptive Coping Strategies; AOP, Adult‐Onset Psychosis; ARMS, At Risk Mental State; ATPD, Acute and Transient Psychotic Disorder; BD, Bipolar Disorder; CHR, Clinical High Risk; CI, Confidence Interval; DBT, Dialectical Behaviour Therapy; DSM, Diagnostic and Statistical Manual of Mental Disorders; EFC, Emotion Focused Coping; ER, Emotional Regulation; F, Female; FDR, First Degree Relative; FEP, First Episode Psychosis; FHR‐P, Familial High Risk of Psychosis; HCs, Healthy Controls; M, Male; MCS, Maladaptive Coping Strategies; NA, Negative Affect; ONPD, Other Nonorganic Psychotic Disorder; OR, Odds Ratio; PA, Positive Affect; PD, Psychotic Disorder; PDD, Persistent Delusional Disorder; PFC, Problem Focused Coping; P‐NOS, Psychosis – not otherwise specified; PTSD, Post‐Traumatic Stress Disorder; SD, Standard Deviation; SZ, Schizophrenia; SZ‐AR, Acutely Relapsed Schizophrenia; TD, Typically‐Developing Adolescents and Young Adults; UHR, Ultra High Risk; UNP, Unspecified Nonorganic Psychosis.

### 3.1 Risk of Bias and Certainty Assessment

According to the EPHPP tool, the global quality rating for the included studies was as follows: ‘strong’ (*n* = 34), ‘moderate’ (*n* = 38), and ‘weak’ (*n* = 4) (see Table 2). Additionally, the two included qualitative studies scored a global rating of ‘strong’ according to the JBI tool (see Table 3). This suggests that the majority of the studies included in the present review included low risk of bias studies.

### 3.2 Emotional Functioning

Six studies examined emotional functioning difficulties in SZ and CHR (Beals et al. 2024; Kimhy et al. 2012; Kimhy et al. 2016; Tarrier et al. 2007; Vines et al. 2021; Visser et al. 2018). Compared to Healthy Controls (HCs), individuals with SZ (Beals et al. 2024; Kimhy et al. 2012; Kimhy et al. 2016) and those at CHR (Kimhy et al. 2016; Vines et al. 2021) demonstrated significant impairments in emotional awareness, emotional understanding of both self and others, and emotional regulation. Additionally, CHR (Vines et al. 2021; Palmier‐Claus et al. 2012; van der Steen et al. 2017) and SZ individuals (Dokuz et al. 2022; Myin‐Germeys et al. 2001) experienced significantly heightened emotional reactivity. SZ also demonstrated significant difficulties in differentiating between positive and negative emotions (i.e., exhibited low emotional granularity) compared to controls but demonstrated an intact ability to differentiate between negative emotions (Kimhy et al. 2014). These collective findings highlight that SZ and CHR exhibit difficulties in managing and understanding their emotions compared to HCs. Lower emotional awareness, negative emotional reactivity, and emotional dysregulation were significantly associated with increased positive symptoms in SZ (Beals et al. 2024; Visser et al. 2018). Additionally, emotional dysregulation and lowered emotional awareness were significantly associated with impaired social functioning in SZ and CHR (Kimhy et al. 2012; Vines et al. 2021). These findings indicate that emotional functioning deficits are relevant across early and established phases of psychosis, impacting positive symptom severity, as well as social outcomes.

### 3.3 Positive and Negative Affect

Eight studies investigated the levels of Positive Affect (PA) and/or Negative Affect (NA) in psychosis (Ader et al. 2024; Grove et al. 2016; Ludwig, Mehl, Krkovic, and Lincoln 2020; Ludwig, Mehl, Schlier, et al. 2020; Moran et al. 2018; Myin‐Germeys et al. 2005; Painter et al. 2019; Yee et al. 2019). Compared to controls, individuals with SZ or Psychotic Disorders (PDs) demonstrated significantly elevated levels of NA (Ader et al. 2024; Grove et al. 2016; Ludwig, Mehl, Schlier, et al. 2020; Myin‐Germeys et al. 2005; Painter et al. 2019; Yee et al. 2019) and significantly reduced levels of PA (Ader et al. 2024; Ludwig, Mehl, Schlier, et al. 2020; Myin‐Germeys et al. 2005; Painter et al. 2019; Yee et al. 2019). NA was a significantly strong predictor of paranoia in PDs, suggesting that managing negative emotions could be crucial in mitigating paranoid symptoms (Ludwig, Mehl, Krkovic, and Lincoln 2020). In PDs, emotional awareness significantly reduced the association between NA and paranoia, whereas rumination strengthened the affective pathway to paranoia, suggesting that difficulties in emotional regulation may exacerbate paranoid symptoms (Ludwig, Mehl, Krkovic, and Lincoln 2020). The habitual use of the coping strategy reappraisal significantly increased PA (Ludwig, Mehl, Schlier, et al. 2020; Moran et al. 2018) and decreased NA (Ludwig, Mehl, Schlier, et al. 2020). However, another study found that the use of reappraisal did not significantly decrease NA (Moran et al. 2018). This negative finding might be attributed to the use of only four distinct emotions (happy, calm, sad, and anxious), which was combined into composite measures of PA and NA (Moran et al. 2018), while the positive finding was derived from a study that operationalised NA and PA using a broader range of eleven emotions (sad, anxious, guilty, irritable, lonely, insecure, nervous, satisfied, happy, relaxed and proud), which may be deemed a more comprehensive measurement of positive and negative affect (Ludwig, Mehl, Schlier, et al. 2020). In contrast to reappraisal, two studies indicated that greater use of the coping strategy suppression significantly reduced PA and increased NA (Ludwig, Mehl, Schlier, et al. 2020; Moran et al. 2018). These findings highlight that reappraisal functions as an ACS, as it tends to decrease NA and increase PA, while suppression acts as an MCS, reducing PA and increasing NA.

### 3.4 Maladaptive vs. Adaptive Coping

Nine studies investigated the role of Maladaptive Coping Strategies (MCS) and Adaptive Coping Strategies (ACS) in individuals with SZ (Holubova et al. 2015; Liu et al. 2020; Meyer 2001; Moran et al. 2018; Moritz et al. 2016; Perry et al. 2011; Piotrowski et al. 2019; Visser et al. 2018; Strauss et al. 2019), and six studies examined these strategies in CHR (Jalbrzikowski et al. 2014; Kang et al. 2018; Masillo et al. 2012; Kommescher et al. 2014; Kimhy et al. 2016; Strauss et al. 2019). Both SZ (Holubova et al. 2015; Meyer 2001; Moritz et al. 2016; Strauss et al. 2019) and CHR individuals (Jalbrzikowski et al. 2014; Kang et al. 2018; Kommescher et al. 2014; Strauss et al. 2019) demonstrated a significantly greater use of MCS than ACS compared to HCs. This signifies that the tendency to rely on MCS may begin before the onset of psychosis. MCS were significantly associated with increased positive and negative symptoms in both CHR and SZ (Jalbrzikowski et al. 2014; Moritz et al. 2016), as well as with lower QoL in SZ (Holubova et al. 2015). Specifically, avoidance was associated with increased negative symptoms, affective flattening, avolition, social withdrawal, and depression severity in SZ (Moritz et al. 2016). Alternatively, ACS were associated with decreased negative symptoms and improved social functioning in CHR (Jalbrzikowski et al. 2014), as well as decreased depression and negative symptom dimensions such as affective flattening, avolition, and social withdrawal in SZ (Moritz et al. 2016). Similarly, greater ACS use was associated with higher QoL in SZ (Holubova et al. 2015). Additionally, CHR individuals tended to employ escape/avoidance coping more frequently than HCs, which were related to negative self‐representations and emotional regulation difficulties (Masillo et al. 2012). In addition, individuals with SZ reported significantly greater use of MCS, such as distraction and situation modification compared to HCs (Piotrowski et al. 2019; Visser et al. 2018). The use of distraction was significantly associated with depressive symptom severity (Piotrowski et al. 2019). Furthermore, MCS such as catastrophic thinking, rumination, self‐blame, and emotional dysregulation were strongly associated with an increase in positive and negative symptoms in SZ (Liu et al. 2020). Conversely, several ACS were associated with beneficial outcomes. Acceptance was significantly associated with reduced depression and improved social functioning (Perry et al. 2011), and the savouring of positive emotions was significantly associated with reduced negative symptoms, enhanced emotional expression, and increased social engagement in SZ (Moran et al. 2018).

Collectively, these findings highlight that individuals with psychosis and those at risk tend to rely more on MCS, which may exacerbate positive and negative symptoms, increase depression, and reduce social functioning. In contrast, ACS appears to support emotional regulation, reduce positive and negative symptoms and improve social functioning. These patterns underscore the importance of promoting ACS and addressing MCS in early intervention treatment efforts.

It is important to note that the majority of studies examining adaptive versus maladaptive coping strategies (ACS vs. MCS) were cross‐sectional. While such designs can identify associations between variables, they preclude causal inferences from being made. Only two studies employed a longitudinal design ranging from 6 weeks in SZ (Meyer 2001) to 12 months in CHR individuals (Jalbrzikowski et al. 2014). In both studies, baseline coping strategies did not significantly predict symptom severity over time. However, this may be due to reduced sample sizes at follow‐up due to attrition, potentially rendering these studies underpowered to detect significant coping‐by‐time interactions. These limitations therefore underscore the need for longitudinal studies to explore the temporal impact of coping strategies on symptom trajectories in psychosis.

### 3.5 Suppression vs. Reappraisal

Ten studies investigated the role of suppression and reappraisal in psychosis (Grove et al. 2016; Henry et al. 2008; Kimhy et al. 2012; Kimhy et al. 2016; Ludwig, Mehl, Schlier, et al. 2020; Moran et al. 2018; Moritz et al. 2016; Perry et al. 2011; Vines et al. 2021; Visser et al. 2018). Compared to HCs, PD and SZ demonstrated a significantly higher use of suppression (Kimhy et al. 2012; Ludwig, Mehl, Schlier, et al. 2020; Visser et al. 2018) and significantly lower use of reappraisal (Kimhy et al. 2012; Ludwig, Mehl, Schlier, et al. 2020; Vines et al. 2021). However, one study reported that SZ used reappraisal more frequently than HCs (Visser et al. 2018), while two other studies found no significant group differences in either reappraisal (Henry et al. 2008; Perry et al. 2011) or suppression (Henry et al. 2008; Perry et al. 2011; Vines et al. 2021). This discrepancy may be attributed to individuals with SZ being in a stable phase of the illness, suggesting that ACS, such as reappraisal, could be learned over time (Henry et al. 2008; Perry et al. 2011; Visser et al. 2018). Additionally, the sample in Vines et al.'s (2021) study consisted of individuals at CHR, indicating that the use of suppression may potentially increase following the onset of psychosis. The use of suppression was significantly associated with increased positive symptoms (e.g., paranoia, bizarre experiences, hallucinations and magical thinking), negative symptoms (e.g., affective flattening, avolition and social withdrawal), and greater depression severity in SZ (Moritz et al. 2016). Suppression was also significantly associated with lower levels of social interaction and interest in SZ (Moran et al. 2018), and poorer social functioning in the CHR group (Kimhy et al. 2016). Conversely, in SZ, the use of reappraisal was significantly associated with reduced overall negative symptoms (Perry et al. 2011), depression (Henry et al. 2008; Perry et al. 2011), and social functioning difficulties (Henry et al. 2008), whereas lower use of reappraisal was significantly associated with greater negative symptom severity in CHR (Vines et al. 2021). These findings highlight that suppression is associated with increases in positive and negative symptoms, as well as social functioning deficits in SZ, whereas reappraisal is associated with improvements in negative symptoms, depression and social functioning. Thus, enhancing reappraisal while reducing suppression may improve clinical outcomes in SZ. However, three studies found no significant association between suppression and positive symptoms (Moran et al. 2018), negative symptoms (Grove et al. 2016; Henry et al. 2008; Moran et al. 2018), or social functioning (Moran et al. 2018), while one study indicated that reappraisal was not associated with positive or negative symptoms (Moran et al. 2018). These differences in findings might be accounted for by the employment of differing measures across studies; for example, the negative findings regarding symptom dimensions with suppression or appraisal employed the Brief Psychiatric Rating Scale (BPRS) and the Scale for the Assessment of Positive Symptoms (SAPS), while the positive findings employed the Community Assessment of Psychic Experiences‐Scale (CAPES) and the Scale for the Assessment of Negative Symptoms (SANS). The CAPES assesses three domains of positive symptomatology, namely, paranoid ideation, bizarre experiences, and perceptual anomalies, while the SANS assesses five domains of negative symptoms including alogia, affective flattening/blunting, anhedonia‐asociality, avolition‐apathy, and attention. In contrast, the BPRS is comprised of 18 items including emotions (anxiety, guilt, hostility, excitement and somatic concern), subjective observations (mannerisms, tension, posturing and uncooperativeness), as well as psychosis symptoms (grandiosity, conceptual disorganisation and hallucinatory behaviour), while the SAPS assesses four domains consisting of delusions, hallucinations, positive formal thought disorder and bizarre behaviour. Therefore, it is possible that suppression and reappraisal may relate to some symptom dimensions more than others, hence the observation of mixed findings across studies. The mixed findings regarding social functioning might also be accounted for by the inclusion of differing social functioning measures across studies. For example, the negative finding employed the Specific Level of Functioning Scale (SLOF), which assesses functioning in interpersonal relationships, participation in community, and work activities, while the positive finding employed the Global Functioning Scale:Social (GFS:S), which assesses peer relationships in terms of age‐appropriate social contacts, romantic relationships and relationship conflict. Therefore, it is possible that suppression might relate more to the items of the GFS:S than the SLOF.

Another factor that may influence individuals' use of suppression and reappraisal pertains to meta‐emotional beliefs. Compared to HCs, individuals with SZ (*n* = 38) reported holding beliefs about emotions as uncontrollable, which were associated with a greater use of suppression, less use of reappraisal, and a greater severity of positive and negative symptoms and depression (Berglund et al. 2023). These authors thus concluded that uncontrollability beliefs could be viable targets for psychological intervention.

### 3.6 Emotion‐Focused vs. Problem‐Focused Coping

Four studies investigated Emotion‐Focused Coping (EFC) versus Problem‐Focused Coping (PFC) in psychosis (Lee and Schepp 2010; Piotrowski et al. 2019; Ritsner et al. 2006; Rudnick 2001). Both SZ and FEP groups demonstrated significantly greater use of EFC than PFC (Lee and Schepp 2010; Piotrowski et al. 2019; Ritsner et al. 2006). EFC was significantly associated with depressive symptom severity (Piotrowski et al. 2019), emotional irritability, cognitive impairment and stress (Lee and Schepp 2010). In contrast, PFC was significantly associated with improved anxiety, social support and personal control (Lee and Schepp 2010). However, one study found no association between PFC or EFC and positive symptoms (Rudnick 2001). This non‐significant finding may be attributed to that study's focus on coping specifically in relation to symptoms (Rudnick 2001), whereas other studies assessed coping across a broader range of contexts, including challenging situations, stressful events and upsetting circumstances. Overall, these findings indicate that EFC is prevalent in SZ and FEP and is associated with negative outcomes, whereas PFC is associated with improved outcomes. Additionally, regression analyses revealed that depressive symptoms and objective QoL predicted PFC, accounting for 32% of the variance, while negative symptoms, self‐esteem, subjective QoL and awareness of psychosis symptoms predicted EFC, accounting for 60% of the variance (Zappia et al. 2012). These authors thus concluded that as these clinical and functional variables influence coping style, they could be clinical intervention targets in CBT to promote more adaptive coping strategies (Zappia et al. 2012).

### 3.7 Anxiety, Depression and Positive Symptoms

Two studies investigated the role of anxiety and depression on positive symptoms (Smith et al. 2006; Thewissen et al. 2011). Individuals with SZ who had higher levels of depression and lower self‐esteem experienced more severe and distressing auditory hallucinations with intensely negative content (Smith et al. 2006). In addition, individuals with SZ who reported higher levels of depression, lower self‐esteem, and more negative self and negative other evaluations experienced more severe persecutory delusions, with greater preoccupation and distress (Smith et al. 2006). Grandiose delusion severity was negatively correlated with depression and negative self‐evaluations and positively correlated with self‐esteem (Smith et al. 2006).

In terms of persecutory delusions and emotions, increased anxiety coupled with decreased self‐esteem predicted the onset of persecutory episodes (Thewissen et al. 2011). Cross‐sectionally, persecutory episodes were associated with low self‐esteem and elevated levels of all negative emotions (Thewissen et al. 2011). The initial intensity of depression and paranoia predicted longer episode duration, whereas anger and irritability were associated with shorter episodes (Thewissen et al. 2011). Overall, these findings highlight the complex interplay between mood, self‐esteem and positive symptoms in SZ and PDs, with depression, anxiety and self‐esteem emerging as key factors influencing the severity and duration of delusions and paranoid episodes.

### 3.8 Interventions

Five studies examined emotion regulation interventions in individuals with SZ (Favrod et al. 2019; Kommescher et al. 2014; Lawlor et al. 2022; Ryan et al. 2021; Yan Lam et al. 2020). An intervention designed to reduce anhedonia and apathy while enhancing positive emotional experiences resulted in significant improvements in emotional experiencing and expression, present‐moment joy, and social functioning (Favrod et al. 2019). Dialectical Behavioural Therapy (DBT) based group interventions were effective in reducing emotional regulation difficulties, enhancing adaptive emotional regulation skills, and were associated with decreased severity of hallucinations, delusions and overall distress (Lawlor et al. 2022; Ryan et al. 2021). Integrated Psychological Intervention (IPI) targeting emotion regulation and coping styles using Cognitive Behavioural Therapy (CBT), group skills training, cognitive remediation, and psychoeducational multifamily groups (to improve familial understanding of psychosis and reduce interpersonal conflict) significantly improved positive symptoms, negative symptoms, and social functioning (Kommescher et al. 2014). Additionally, a mindfulness‐based group intervention significantly reduced rumination across time and improved levels of reappraisal at 3‐month follow‐up in individuals with SZ (Yan Lam et al. 2020). There were also significant improvements in the overall level of psychosis symptoms and hallucinations, including their physical characteristics, controllability and cognitive interpretation (Yan Lam et al. 2020).

It is important to note that not all interventions explicitly targeted emotional functioning or emotion regulation as a central mechanism of change. While DBT‐based interventions directly targeted emotional regulation difficulties, other interventions such as CBT, cognitive remediation or psychoeducational multifamily groups may have led to emotional improvements indirectly. These effects could stem from targeting associated factors such as negative cognitions, positive symptoms, or improved family understanding and social support. This distinction highlights the need for further research to clarify whether improvements in emotional functioning result from directly targeting emotion regulation processes and emotional coping or emerge secondarily through broader therapeutic mechanisms.

### 3.9 Qualitative Study

Only one qualitative study to date has explored the subjective experience of emotions before, during, and after an episode of psychosis in a FEP sample (Hutchins et al. 2016). Participants reported feeling intense emotions such as anger, guilt, fear and sadness, which appeared to be amplified during the psychosis episode. Prior to the episode, participants employed various strategies to manage their emotional intensity, including rumination and suppression to avoid their emotions. However, during the episode, these strategies were less apparent, and emotions felt all‐encompassing. After the episode, participants reported increased feelings of responsibility, self‐criticism, despair, along with a change in their self‐perception. These authors suggest that emotional avoidance or preoccupation may contribute to difficulties in emotional regulation, potentially influencing the onset and persistence of psychosis. These findings highlight the central role of emotions in the experience of psychosis.

### 3.10 Ecological Momentary Assessment (EMA) Studies

Evidence from EMA studies has demonstrated that emotional instability and elevated negative affect are positively associated with momentary experiences of paranoia in individuals with psychotic disorders (Nittel et al. 2018; Orth et al. 2022). Negative affect has also been shown to mediate the relationship between stress and psychosis symptoms, suggesting that stress impacts psychosis via an affective pathway (Klippel et al. 2022). Additionally, the use of expressive suppression in response to negative emotions was found to predict subsequent increases in state paranoia (Nittel et al. 2018) and was associated with heightened momentary experiences of thought insertion, mind reading, and hallucinations (Kimhy et al. 2020). Difficulties in identifying emotions, an aspect of impaired emotional awareness, further moderated the impact of emotion regulation strategies on psychosis symptoms (Kimhy et al. 2020). Collectively, these findings highlight the role of emotional instability, maladaptive emotion regulation, and poor emotional awareness in the expression of psychosis and support targeting these mechanisms in interventions such as CBT for psychosis.

Compared to controls, individuals with SZ exhibited a low threshold for identifying the need to regulate, tending to over‐regulate when NA was low and under‐regulate when NA was high (Raugh and Strauss 2022; Visser et al. 2018). Regulation effort was similarly inefficient, with excessive effort at low NA levels and insufficient effort at high NA levels (Raugh and Strauss 2022; Visser et al. 2018). Despite exerting greater effort, emotion regulation was often ineffective. Compared to controls, individuals with SZ tended to select more strategies that were often not contextually appropriate (Visser et al. 2018). These findings suggest that early‐stage identification deficits may disrupt later stages of regulation and contribute to symptom expression, highlighting a potential target for intervention (Raugh and Strauss 2022; Visser et al. 2018).

Individuals with SZ also demonstrated excessive switching between emotional regulation strategies and delayed stopping compared to controls (Bartolomeo et al. 2022). The authors suggest that this may reflect difficulty in identifying and selecting contextually appropriate strategies (Bartolomeo et al. 2022). As a result, they may frequently shift between ineffective strategies and spend more time attempting to regulate their emotions (Bartolomeo et al. 2022). It is important to note that the specific strategies selected and switched between were not examined; therefore, the identification of specific strategies in relation to specific contexts is worthy of further quantitative and qualitative investigation across the psychosis spectrum (CHR, FEP and SZ). This study also found symptom differences regarding switching frequency and time spent regulating emotions (Bartolomeo et al. 2022). Reduced switching frequency was significantly correlated with negative symptoms (avolition and asociality) but not positive symptoms of psychosis (Bartolomeo et al. 2022). Additionally, negative symptoms were significantly correlated with less time spent regulating one's emotions, while positive symptoms (more severe momentary delusions) were significantly correlated with higher rates of continuing to regulate. Therefore, negative symptoms may reduce one's persistence in emotion regulation attempts, while positive symptoms may make an individual more likely to persist. These findings highlight symptom‐specific differences in emotional regulation in SZ. It is important to note that these findings were derived from a sample of stable outpatients with SZ, so their applicability to acute phases of psychosis, such as those seen in inpatient settings or during a first episode of psychosis, remains unclear. Additionally, although switching and stopping were examined in the present study, another monitoring dynamic, pertaining to *maintenance* (defined as the effective implementation of an emotional regulation strategy for a given context, Bartolomeo et al. 2022), was not investigated and thus highlights avenues for future research. These authors thus suggested that as EMA can investigate the temporal sequence of emotional regulation strategies and their effectiveness over time, it is well suited to elucidating maintenance dynamics in SZ (Bartolomeo et al. 2022).

EMA data has also been used to explore the emotional reactivity and regulation in response to the presence or absence of psychosis (Strauss et al. 2019). The presence of visual hallucinations was associated with the effortful use of distraction, soothing, suppression, and situation modification, while there were no differences for reappraisal or interpersonal strategies (Strauss et al. 2019). Auditory hallucinations were associated with suppression and soothing, while paranoia was associated with the greater use of reappraisal, distraction, suppression and soothing (Strauss et al. 2019). Situation modification was attempted more frequently in response to negatively valenced visual hallucinations compared to benign ones, whereas both situation modification and distraction were employed for auditory hallucinations (Strauss et al. 2019). Participants were found to endorse a significantly higher total number of strategies at each assessment during periods of active psychosis (Strauss et al. 2019). These authors therefore suggest that individuals with psychosis appear to be selecting too many strategies rather than too few that were not always contextually appropriate or adaptive (Strauss et al. 2019).

These authors noted some limitations, including that the small number of EMA surveys captured in the present study prohibited probabilistic chain analysis from being conducted, which would have allowed for the exploration of the temporal sequence of events regarding affective state, the subsequent selection of an emotional regulation strategy, and the outcome of this choice of selection (i.e., whether it lessened the intensity of the negative affect that prompted the emotional regulation strategy in the first instance). Future investigations would therefore benefit from building on these preliminary findings by employing a higher number of EMA surveys (e.g., 10–12), which would provide more power to examine the temporal dynamics of emotional regulation strategies in psychosis (Strauss et al. 2019). Second, the authors note that, as the sample studied outpatients with chronic schizophrenia or schizoaffective disorder, this highlights that the present findings may not be generalisable to individuals at CHR, FEP or those within an acute inpatient setting (Strauss et al. 2019). Third, as common psychosis symptoms were examined in the present study, it remains unclear how emotion is regulated in individuals presenting with other delusional subtypes (i.e., guilt, grandiosity) and hallucinatory subtypes (i.e., olfactory, tactile and gustatory), which requires further exploration (Strauss et al. 2019). Lastly, as the EMA data was ascertained via self‐report, it is subject to social desirability bias. Future studies would benefit from pairing EMA data with psychophysiological recordings during real‐world contexts via mobile technology, which would allow for the elucidation of the mechanistic understanding of emotion in psychosis (Strauss et al. 2019).

### 3.11 Trauma and Emotional Vulnerability

Trauma has been found to influence emotional processes in individuals with psychosis (Stanton et al. 2020).

Childhood trauma such as physical, sexual, emotional abuse and physical/emotional neglect have been associated with heightened stress sensitivity (Lardinois et al. 2011; Reininghaus et al. 2016), elevated stress reactivity (Myin‐Germeys and van Os 2007; Dokuz et al. 2022), reduced emotional awareness and expression (Beals et al. 2024; Lysaker et al. 2011), and emotional dysregulation (Lincoln et al. 2017; Liu et al. 2020). Emotional dysregulation and maladaptive emotional coping have also been found to mediate the relationship between childhood trauma and positive symptoms of psychosis (Liu et al. 2020). It is possible that early adversity may arrest emotional development, thereby reducing emotional regulation capabilities and increasing the likelihood of engaging in maladaptive coping strategies such as suppression or rumination. These strategies have been shown to intensify negative affect and disrupt emotional processing (Gross and Jazaieri 2014; Nolen‐Hoeksema et al. 2008). In psychosis‐prone individuals, sustained emotional distress may alter cognitive appraisals and perceptual salience, increasing the likelihood of misinterpreting internal experiences as externally caused, thereby contributing to the emergence of anomalous experiences.

### 3.12 Summary

These findings provide consistent evidence of emotional difficulties across the psychosis continuum, including impairments in emotional functioning, elevated negative affect and greater reliance on maladaptive coping strategies. Childhood trauma appears to contribute to psychosis risk by disrupting emotional development and promoting maladaptive affect regulation. Individuals at CHR, FEP and those with SZ demonstrated greater use of maladaptive coping strategies such as suppression and avoidance, which are associated with increased symptom severity and poorer social functioning. Conversely, adaptive strategies such as reappraisal and problem‐focused coping show potential protective effects, including symptom reduction and improvements in QoL. The overall body of evidence underscores the critical role of emotion and emotional coping in the onset and persistence of psychosis. These findings provide a foundation for theoretical models that account for these emotional processes and inform the development of targeted interventions.

## 4 Discussion

**FIGURE 2:**
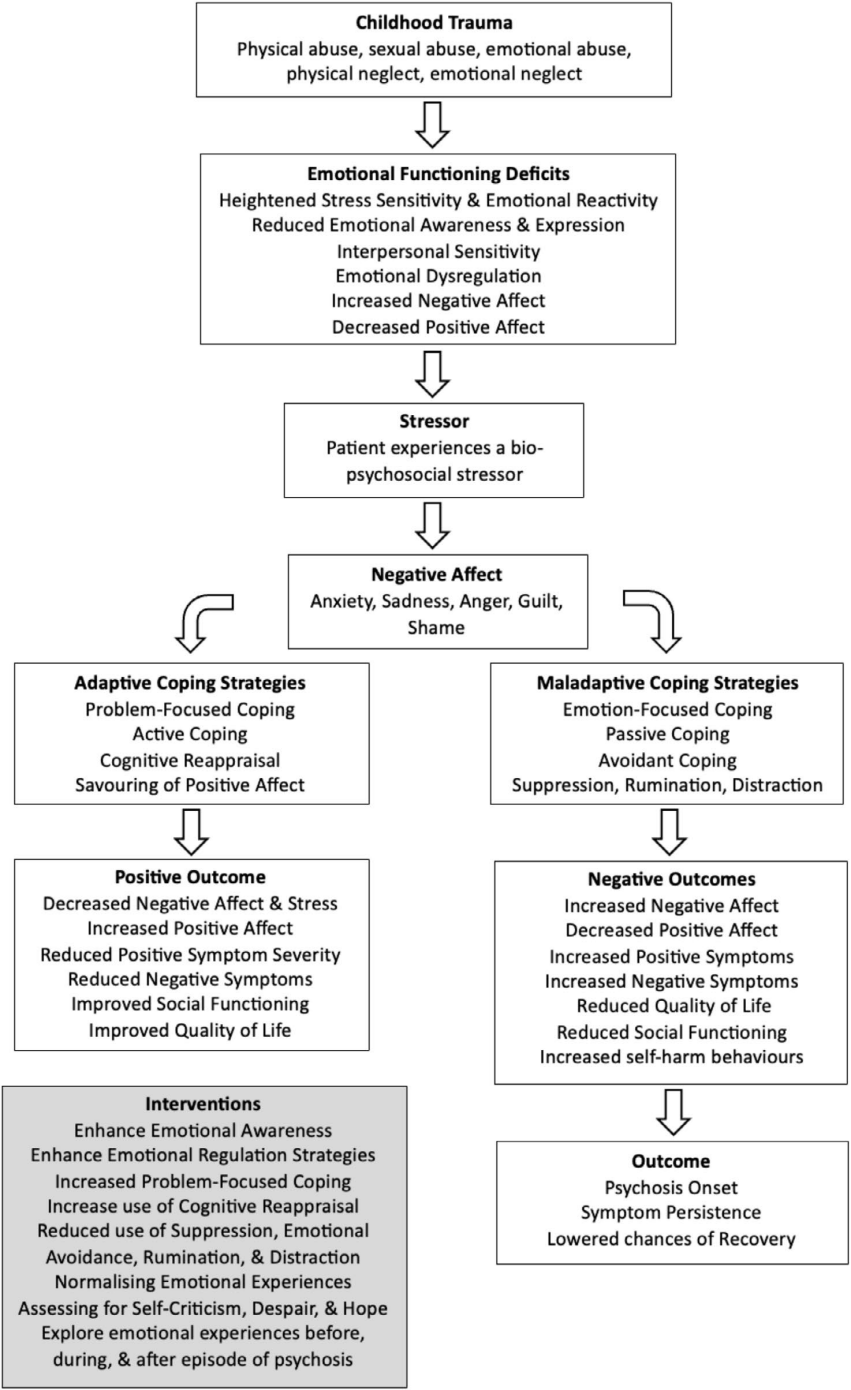
Emotion coping model for psychosis—A conceptual framework illustrating the outcomes of adaptive versus maladaptive coping strategies in psychosis.

Based on the findings of the present review, we propose the following *Emotion Coping Model for Psychosis* (see Figure 2). Our model proposes that childhood traumatic events such as physical/sexual/emotional abuse and physical/emotional neglect, contribute to emotional functioning deficits such as heightened stress sensitivity and emotional reactivity, reduced emotional awareness and expression, and emotional dysregulation. A bio‐psychosocial stressor subsequently experienced would then induce a state of high NA such as anxiety, sadness, anger, guilt or shame. The type of emotion coping strategy employed in response will, in turn, influence clinical outcomes. MCS encompasses the use of emotion‐focused, passive, and avoidant coping, as well as suppression, rumination, and distraction, which would lead to negative outcomes such as further exacerbations of NA, a decrease in PA, and an increase in positive and negative symptomatology. This may subsequently lead to psychosis onset, symptom persistence, and lowered chances of recovery. Alternatively, ACS encompasses problem‐focused and active coping, cognitive reappraisal and the savouring of PA. These strategies reduce the intensity of NA, enhance the experience of PA and lessen the severity of both positive and negative symptoms. Therefore, interventions that enhance emotional awareness, emotional regulation, PFC and cognitive reappraisal, while reducing EFC, rumination, suppression and avoidance may facilitate more adaptive responses to NA, which in turn may reduce psychosis onset and symptom persistence.

While this model depicts two broad coping pathways in psychosis (adaptive vs. maladaptive) grounded in empirical evidence, it is important to note that coping is momentary and context dependent. Additionally, individuals with psychosis may employ multiple coping strategies across situations and time points, a process known as polyregulation. Clinical exploration should therefore assess coping flexibility, context and both short‐ and long‐term outcomes of emotion regulation choices. This clinical heuristic model serves as a practical framework to facilitate therapeutic discussions about emotional coping. It enables clinicians to identify common emotional coping patterns and their associated outcomes, thereby supporting the functional analysis of habitual emotion regulation strategies in psychosis. Clinicians could therefore conduct a functional analysis of the client's emotional experiences by examining antecedents, beliefs, emotions and coping strategies across different contexts. Examining the client's emotion‐coping choices in specific situations allows for a discussion of their short‐ and long‐term advantages and disadvantages (Troy et al. 2023). This approach acknowledges that certain emotion regulation strategies may provide temporary relief (i.e., functionally adaptive in the short term) and emphasises the importance of exploring these strategies not from a deficit approach, but in terms of their contextual utility and long‐term consequences. Unhelpful coping strategies may provide temporary relief from unpleasant emotional stimuli, which becomes negatively reinforced and thereby habituated into patterns of recurring behaviour. However, the exploration of any negative longer‐term effects would be helpful; for example, suppression may help to avoid negative affect in the short term but avoids the processing of emotions, which may inadvertently increase exacerbations of emotionality and/or risk of psychosis relapse. Additionally, clients may hold positive beliefs about rumination (i.e., ‘spending hours thinking about the same situation will help me to solve my problems’); however, ruminative processes serve to maintain high levels of negative affect (depression and/or anxiety) owing to its associated lack of active problem‐solving. Therefore, the advantages and disadvantages of each coping method employed should be explored to encourage more adaptive ways of coping. Table 5 presents Socratic questions to explore emotions and emotional coping in psychosis, whereas Table 6 offers a clinical tool designed to facilitate the functional analysis of emotion coping strategies in this population.

**TABLE 5:** Socratic questions exploring emotions and emotional coping in psychosis.

**Chain Analysis** What situations tend to trigger intense feelings? What happens first? Then what happens next? And then what happens? Can we break down the sequence of events and your reactions step‐by‐step?
**Identification of Emotions** Can you identify what emotions you are feeling right now? What emotions were you feeling during [the incident]? And what emotions were you feeling afterwards? Where do you notice these emotions in your body? Can you identify what emotions someone else is feeling?—Discuss recognising emotions in others and the value of this for social connection, provide psychoeducation about emotions
**Negative Affect** What difficult emotions do you tend to experience? (e.g., anxiety, sadness, anger, guilt, shame and disgust) What tends to trigger these negative emotions? [explore context] What emotion or emotions were particularly strong in the 1‐2 months *before* your psychosis episode/hospitalisation? What was the predominant emotion you were experiencing *during* your episode/when you were in hospital? (e.g., anxiety due to persecution/sadness in response to hearing critical voices) And what was the predominant emotion you were feeling *after* your psychosis episode? (e.g., embarrassment for the loss of control and disruption psychosis caused/continued feelings of anxiety that some threat of harm will occur)
**Emotional Coping (adaptive vs. maladaptive)** When you feel emotionally overwhelmed, how do you tend to cope, what do you do? [Socially withdraw/isolate/act confrontationally/irritably/use alcohol/substances/overthinking/block out emotions] What helps when you are emotionally overwhelmed and what doesn't help?—Explore helpful and unhelpful emotional coping, encourage more helpful coping and the reduction/cessation of unhelpful coping How do you tend to cope with negative emotions (sadness/anger/anxiety/guilt/shame/disgust)?—Explore how negative affect is responded to [negative affect is avoided versus mindfully acknowledged and processed/used as a source of information] Do you tend to feel these difficult emotions, or do you try to avoid them?—Discuss advantages and disadvantages of emotional avoidance/suppression, may be helpful in the short term but may increase psychosis emergence in the long‐term, need to process emotions rather than suppress them/bottle them up What are the short‐ and long‐term consequences of this emotion coping method? (e.g., it provides temporary relief but makes the suspiciousness/voices worse in the long term) Are there any emotion coping strategies you haven't explored yet, like changing how you think about the situation, problem‐solving, distraction or expressing your thoughts and feelings to someone you trust?
**Suppression** When you feel strong emotions, do you try to hide them or push them away? What do you think will happen when you suppress your feelings? And what actually happens? Does suppressing how you feel help in the moment? What happens later? Have you ever felt more distressed after trying to suppress or block out your emotions? What are the advantages and disadvantages of suppressing your emotions?
**Reappraisal** Have you ever tried viewing a stressful situation differently, maybe by seeing it as temporary or as less personal? Can you think of a time when changing how you thought about something helped you to feel better? What would someone you trust say about this situation? Could their perspective help you to see it in a different way? If this were happening to someone else, how would you help them think about it? What could be an alternative possible explanation for what has happened, one that is less distressing?
**Distraction** When you are overwhelmed, do you try to take your mind off it with an activity or task? What kinds of things help you shift your focus when you are feeling low or anxious? How long does the relief from distraction usually last? Does it ever feel like you are avoiding dealing with the emotion by distracting yourself? In what situations can distraction be helpful for you and are there times when it keeps you feeling stuck?
**Positive Affect** What makes you feel positive emotions?—Explore thoughts and behaviours that enhance positive affect What helps you enjoy or amplify these positive feelings? Is this something you can try to do each week?
**Meta‐Emotional Beliefs** How do you tend to view your emotions? [Explore meta‐emotional beliefs‐ dangerous/uncontrollable/enduring] What beliefs do you hold about your emotions (i.e., that they are useful/source of information, dangerous, uncontrollable, enduring, will lead to a relapse if experienced, or will make you lose control/become suicidal?) Can you sit with these emotions and what do you think will happen if you do? (i.e., they will go on indefinitely, will get more intense, will lead you to act in ways you will later regret, or will gradually lessen in intensity?)
**Emotion Regulation Flexibility** Have there been times when a coping strategy that usually helps didn't work as well? What did you do then? (Explores flexibility and willingness to adapt emotion regulation efforts) What might be a helpful way to respond in a similar situation next time, what would you like to try? (Encourages proactive planning and generalisation of new coping skills) Do you notice that you need to use certain coping strategies in response to different symptoms or situations? (e.g., voices, suspiciousness, anxiety, low mood, loneliness, arguments and stress at work)
**Emotion‐Psychosis Associations** Do you notice a connection between how you are feeling emotionally and your voices/suspicious thoughts? Have you noticed if certain emotions come up just before you hear voices/feel suspicious/have distressing thoughts? Do certain emotions make your symptoms worse? Which ones seem to make them better or quieter? What happens to your symptoms when you are feeling emotionally calm or supported? If you could respond differently to the emotion (e.g., anxiety, sadness, anger, guilt, shame and disgust), do you think it would change the intensity or frequency of these experiences? Have you noticed any changes in your voices, suspiciousness, intrusive thoughts, or nightmares when you are able to express or process your emotions more directly?
**Emotion Focused Coping** When something upsetting happens, do you find yourself focusing more on how you feel, rather than trying to solve the problem/address the situation? How do you usually try to soothe or comfort yourself when you are feeling stressed or overwhelmed? (relaxation/meditation/avoidance/social withdrawal/seeking social support/talk to others/positive reframing) Do you tend to keep things to yourself or talk to someone when you are feeling emotional? When you are upset, do you try to calm yourself down, distract yourself, or allow yourself to feel the emotions?
**Evaluating Emotion Focused Coping Strategies** How well do your usual ways of coping with difficult emotions work for you in the short term? And in the long term? Are there certain ways of coping with emotions that feel good in the moment, but leave you feeling worse later? Do you ever avoid situations or feelings because they bring up strong emotions? What is the impact of that? What effect does avoiding your feelings have on your voices/suspiciousness? [symptoms]
**Exploring Alternative Coping Methods** Are there other ways you could respond to emotional distress that might be more helpful? What would it look like to accept or make space for your emotions instead of pushing them away? What helps you feel emotionally safe and grounded when things feel intense?
**Problem solving** Can you think of a problem you would like to solve currently? Can you brainstorm all possible solutions? Let's look at the advantages and disadvantages of each proposed solution and decide which might be worth trying. Can you pick one solution and try it out and see how effective it was?

**TABLE 6:** Functional analysis of emotion coping strategies in psychosis.

Antecedent (and context)	Belief	Emotion (% intensity)	Emotional coping method (Helpful+ vs. Unhelpful−)	Short‐term (ST) versus long‐term (LT) consequences of emotional coping method	New emotional coping Strategies to try
Critical family member (someone that I live with)	Others are hostile	Anger (80%)	Suppress (−)	ST: Helps me to cope with my anger LT: Increases suspiciousness	Mindful acceptance of emotion
Distressing news story (On the TV)	The world is dangerous	Anxiety (70%)	Alcohol (−)	ST: Helps me to sleep LT: Feels worse the next day	Distraction (change channel), talk to family
Argument with friend (Someone that I see every week)	I'm unlikeable	Sadness (60%)	Problem‐solve (+) (text friend to apologise)	ST: Fear of rejection LT: No more guilt because I tried to mend the friendship	Express feelings, validate sadness
Awaiting exam results (Need good grades to get into university)	I must dwell on this uncertainty	Anxiety (90%)	Rumination (−)	ST: Rumination increases my anxiety LT: I feel more anxious	Notice, interrupt, distract Problem‐solve, explore alternative options if grades are not met
Memories of the past (Having nightmares twice a week of past traumas)	I'm worthless and weak	Sadness (90%)	Suppress, distract (−)	ST: Distraction provides temporary relief LT: The nightmares continue and are more intense	Build tolerance to emotion, mindful awareness Use grounding techniques Reappraisal‐ I am not weak
Feeling watched in public (when in the shops)	People are laughing at me	Shame (85%)	Withdraw (−)	ST: Relief from social anxiety LT: Increased isolation	Behavioural experiment: go out, stay with feeling, and notice it rise and naturally reduce over time
Voice says ‘you're bad’ (after argument)	I deserve this punishment	Guilt (75%)	Suppress (−)	ST: Avoid painful feelings LT: Voice becomes more distressing	Self‐soothing, Reappraisal: Challenge belief about badness Talk to therapist
Invited to a party (with friends)	No one will like me	Sadness (70%)	Avoid/Don't attend (−)	ST: Relief from anticipated discomfort LT: Increased social isolation, missed opportunities for connection/positive reward	Attend, use mindful acceptance to sit with the emotion and observe its intensity fade Notice any positive experiences
Anxious before work meeting (with the boss)	Emotions are dangerous and must be avoided, they are a sign that I am relapsing and will end up in hospital [Meta‐emotional beliefs]	Anxiety (90%)	Suppress (−)	ST: I feel temporarily more in control LT: My anxiety builds and becomes harder to manage over time; the voices and suspiciousness also become more intense, reinforces fear about relapse	Emotion labelling (e.g., ‘I'm feeling anxious right now’—name it to tame it), mindful acceptance, psychoeducation about emotions Reappraisal: emotions are not dangerous, they are signals and sources of information, not threats
Felt overwhelmed at work (facing a backlog of emails following a week of annual leave)	‘I can't cope’	Anxiety (60%)	Took a break and used breathing techniques (+) Problem‐solve (+)	ST: Anxiety reduced; clearer thinking LT: Felt more capable managing stress	Continue using breathing and add a walk outside, use active problem‐solving by starting to work through the emails systematically, for example, make a to‐do list prioritising urgent tasks
Received upsetting message (from friend)	‘I'm not good enough’	Sadness (70%)	Spoke with a friend (+)	ST: Felt heard and supported LT: Strengthened relationship and reduced isolation	Schedule regular check‐ins with supportive people
Missed a deadline at work (got the dates mixed up)	‘I've ruined everything’	Shame (65%)	Reappraised the situation (+)	ST: Reduced shame, able to focus on next task LT: Built resilience and problem‐solving mindset	Reappraisal: Remind myself that one mistake doesn't define my capability/entire being Focus on what can be done next
Feeling low after a lonely weekend	‘I'll always be alone’	Sadness (65%)	Chose to message a friend and go for a walk (+)	ST: Mood lifted slightly, felt more connected LT: Built social support, reinforced values of connection and self‐care	Create a routine for reaching out even when feeling down Focus on small, valued actions
Anniversary of a relative's passing (Avoidance of grief)	*‘If I think about them, it will hurt too much’*.	Sadness (80%)	Avoidance of grief rituals (−)	ST: Protects from distress in the moment LT: Grief remains unresolved, and resurfaces intensely during anniversaries or reminders	Mark the day meaningfully: light a candle or write a letter Allow gentle connection with memories (e.g., photo album) Reappraise: Avoidance maintains sadness, acceptance helps the grieving process
Anniversary of a relative's passing (Meta‐emotional fear of sadness)	*‘If I let myself feel this sadness, it will never end and might make me feel suicidal’* [Meta‐emotional belief: Sadness is enduring, dangerous, and uncontrollable]	Anxiety (90%)	Suppress emotions (−) Over‐control (−)	ST: Temporary sense of control LT: Heightened distress and fear of relapse or emotional breakdown	Reappraise: sadness is a normal response to loss, not a danger signal Graded exposure: increase exposure to sadness in small increments Create a safety plan for high distress (who to contact, what helps to regulate my mood)

**FIGURE 3:**
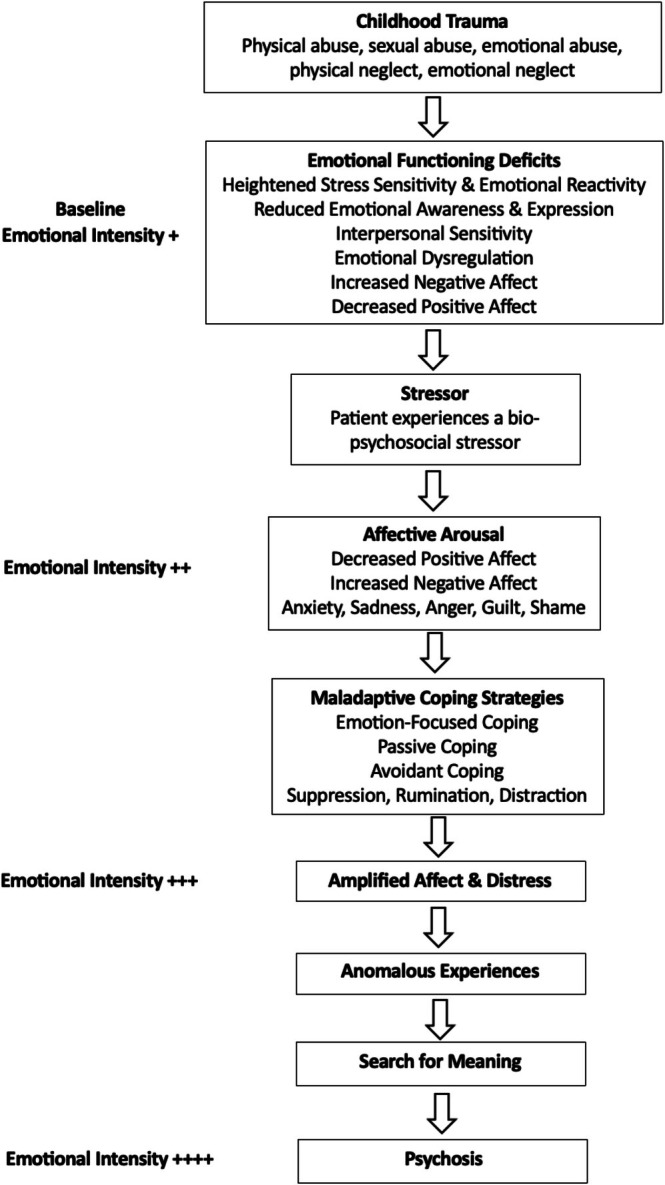
Affective pathway model of psychosis—A conceptual framework for understanding the role of emotions in psychosis.

**FIGURE 4:**
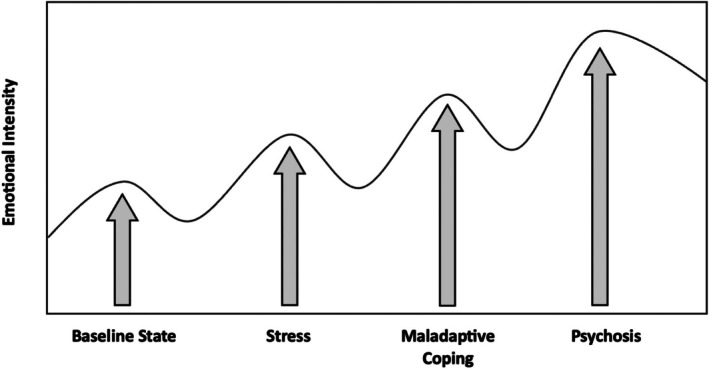
Visual representation of the amplification of affect in the lead‐up to psychosis onset.

Given the prominence of emotion in psychosis, we propose the following *Affective Pathway Model of Psychosis* (see Figure 3). Our model proposes that childhood traumatic events such as physical/sexual/emotional abuse and physical/emotional neglect contribute to emotional functioning deficits such as heightened stress sensitivity and emotional reactivity, reduced emotional awareness and expression, and emotional dysregulation. A bio‐psychosocial stressor subsequently experienced would then induce a state of high NA such as anxiety, sadness, anger, guilt or shame. Although the employment of MCS such as EFC, passive coping, and avoidant coping, as well as the use of suppression, rumination, and distraction may provide temporary relief, they would lead to an amplification of affect and distress. This emotional intensification will serve as an affective pathway to the generation of anomalous experiences. One's search for meaning for internal or external ambiguous stimuli would then either halt the exacerbation of anomalous experiences or magnify them further, leading to the formation of manifest psychosis. This model suggests that an individual with a predisposition to psychosis will have an elevated baseline emotional intensity compared to the general population. This model highlights four sequential peaks in emotional escalation: (1) a heightened baseline emotional intensity due to predisposition to psychosis, (2) further escalation triggered by bio‐psychosocial stressors, (3) additional intensification following the use of MCS, and (4) a final amplification of affect during the episode of psychosis (see Figure 4).

The *Emotion Coping Model* and the *Affective Pathway Model* serve complementary but distinct purposes in understanding emotion in psychosis. The *Emotion Coping Model* focuses on moment‐to‐moment emotion regulation strategies, aiding clinicians in identifying and modifying habitual coping behaviours to promote more adaptive responses and reduce less adaptive ones. In contrast, the *Affective Pathway Model* conceptualises the role of affect in the onset of psychosis, supporting clinicians in developing longitudinal formulations of a client's psychosis emergence and informing relapse prevention. While both models align with frameworks such as Garland et al.'s (2010) upward and downward spiral models, which describe how emotion regulation patterns contribute to either escalating distress or recovery, they operate at different levels of clinical application. The *Emotion Coping Model* addresses current emotion regulation processes, whereas the *Affective Pathway Model* supports a broader understanding of the cumulative role of affect in psychosis onset and symptom persistence. Used in parallel, these models provide clinicians with distinct yet synergistic tools for understanding and addressing emotional processes in psychosis.

While both the *Emotion Coping Model* and the *Affective Pathway Model* emphasise the critical role of emotional processes in psychosis, they differ in their conceptualization of the temporal sequence and mechanisms involved. The *Affective Pathway Model* primarily focuses on the developmental trajectory whereby early trauma and stress lead to heightened emotional vulnerability, which in turn amplifies negative affect and facilitates the emergence of aberrant experiences that may culminate in psychosis onset. This model highlights a macro‐level, longitudinal perspective, where negative affect contributes to symptom development and persistence over time. In contrast, the *Emotion Coping Model* centres on moment‐to‐moment regulation of negative affect, detailing how individuals' habitual coping strategies influence the immediate experience and modulation of distress. This model offers a micro‐level, clinical heuristic that helps clinicians explore how maladaptive coping (e.g., suppression, rumination and avoidance) maintains or exacerbates negative emotions and symptom severity in real time. Importantly, it underscores that while aberrant experiences and negative affect are intertwined, the way in which individuals cope with these emotions can directly impact symptom persistence and recovery pathways. By explicitly focusing on the dynamic interplay of coping behaviours and emotional regulation as they unfold in the present moment, the *Emotion Coping Model* adds to existing conceptualizations of psychosis aetiology by identifying actionable targets for intervention that may interrupt maladaptive emotional cycles before they solidify into chronic symptomatology. This complements the broader longitudinal framework of the *Affective Pathway Model*, enabling a more nuanced understanding of psychosis that integrates both the developmental origins of affective dysfunction and the ongoing processes of emotional coping that influence clinical outcomes. While these models share theoretical roots, maintaining their distinction facilitates targeted clinical interventions addressing both the developmental origins and immediate emotional coping challenges in psychosis.

While trauma is emphasised in the present model due to its well‐documented role in heightening emotional vulnerability and its clinical relevance in psychosis, it is important to recognise that psychosis aetiology is multifactorial. Genetic predispositions and other biological vulnerabilities substantially contribute to risk and interact with environmental factors across development (Zwicker et al. 2018). Additionally, social and environmental influences such as socioeconomic status (Kirkbride et al. 2008), experiences of discrimination (Bardol et al. 2020), cultural norms regarding emotional expression (Safdar et al. 2009), and one's social and physical environment (overcrowding, noise, sense of safety) (Fischer et al. 2003; Zhang et al. 2023) can influence emotional experiences and coping strategies. These factors may modulate the pathways by which emotional dysregulation contributes to psychosis onset and persistence. Integrating these broader influences within the model offers a more comprehensive biopsychosocial understanding of psychosis and highlights the need for holistic clinical approaches addressing biological, psychological and social determinants of emotional functioning and psychosis risk.

While the current model emphasises emotional dysregulation in the pathway between childhood trauma and psychosis risk, it is also possible that core beliefs formed in response to trauma may also contribute to the development of emotional vulnerability. Recent meta‐analytic findings show large effects for negative self‐beliefs in SZ and CHR compared to controls (Jorovat et al. 2025). Additionally, a longitudinal structural equation modelling study in a non‐clinical sample found a significant unidirectional path from negative core beliefs to positive symptoms, and a bidirectional association between negative beliefs and negative affect over time (Jaya et al. 2018). These findings support an affective pathway in which negative beliefs and negative affect reinforce one another, ultimately contributing to psychosis (Jaya et al. 2018). This aligns with the *Bio‐Psychosocial Model of Transition to Psychosis*, which proposes that reactivation of core beliefs in response to psychosocial stressors triggers heightened negative affect, serving as an affective pathway to psychosis onset (Georgiades et al. 2023). Future research could therefore explore whether core beliefs serve as an intermediary between trauma and emotional dysregulation, thereby offering a more comprehensive cognitive‐affective framework for understanding psychosis. Integrating this dimension may help refine existing models and inform cognitive interventions targeting belief systems alongside emotional processing.

This systematic review investigated the role of emotions and emotional coping across the psychosis continuum. Compared to HCs, SZ and CHR demonstrated significant impairments in emotional functioning, emotional awareness and emotional regulation along with heightened emotional reactivity. Lower emotional awareness, heightened negative emotional reactivity and greater emotional dysregulation were significantly associated with increased positive symptoms in SZ, as well as impaired social functioning in both SZ and CHR. SZ and PD reported high levels of NA and low levels of PA, with NA being a strong predictor of paranoia in PDs, and rumination strengthening the affective pathway to paranoia. These findings might be explained by early adverse childhood experiences leading to increased stress sensitivity, emotional reactivity and NA (Kramer et al. 2013). Indeed, trauma has repeatedly been implicated in the onset and exacerbation of psychosis (Stanton et al. 2020; Varese et al. 2012), with 89% of individuals with a FEP reporting one or more adversities compared to 37% of controls (Trauelsen et al. 2015). Childhood maltreatment has been found to cause emotional awareness deficits in SZ (Beals et al. 2024), and emotional regulation difficulties have been found to mediate the relationship between childhood trauma and psychosis symptoms (Lincoln et al. 2017). Enduring childhood traumatic experiences have also demonstrated neurobiological correlates, such as reduced amygdala volume and activity, as well as altered functional connectivity with the prefrontal cortex, a region implicated in psychosis symptomatology (Giannopoulou et al. 2023). Daily stressors have also been found to increase affective arousal, which in turn contributes to psychosis symptoms (Kramer et al. 2013; Myin‐Germeys and van Os 2007). The affective response to these stressors is thought to be influenced by emotion regulation whereby the failure to employ adaptive emotion regulation strategies exacerbates the affective stress response. This heightened stress response subsequently leads to increasingly unbearable NA, thereby elevating the likelihood of beliefs that align with distressing emotions, such as the development of threat beliefs in the context of extreme anxiety (Freeman et al. 2001). Furthermore, the use of MCS, such as suppression, may trigger psychosis symptoms via the misinterpretation of one's affective states, thus increasing the likelihood of persecutory appraisals.

According to the cognitive model of positive symptoms of psychosis (Garety et al. 2001), a triggering event or anomalous experience can result in emotional changes, which feed back into the moment‐by‐moment processing of anomalous experiences and influence their content. Indeed, individuals with SZ who exhibited higher depression and lower self‐esteem experienced more severe and distressing auditory hallucinations with intensely negative content. Moreover, in individuals with SZ, increased depression, lower self‐esteem and negative evaluations about self and others were significantly associated with greater severity of persecutory delusions (Smith et al. 2006). Indeed, depression has been found to contribute to hallucinations and delusions, suggesting that feelings of hopelessness and uncontrollability contribute to symptom maintenance (Birchwood and Iqbal 1998). Consistent with this, hopelessness is a predictor of poor outcomes in psychosis (Aguilar et al. 1997). The cognitive model of psychosis posits that anxiety can maintain psychosis (Garety et al. 2001), whereas safety‐behaviours can prevent the receipt of disconfirmatory evidence and hence prevent change in psychosis appraisals (Garety et al. 2001). Meta‐cognitive beliefs, such as beliefs concerning the uncontrollability of one's thoughts, may further increase the distress caused by psychosis symptoms (Freeman and Garety 1999). Moreover, emotion has been posited to influence one's search for meaning in terms of affect‐associated beliefs, for example, anxiety will increase the probability of a threat belief being chosen.

In terms of emotional coping, SZ and CHR employed MCS significantly more than ACS, and relied more on EFC than PFC, which were associated with worse and better clinical outcomes, respectively. This suggests a pattern of maladaptive coping that may exacerbate emotional distress and contribute to the persistence of both positive and negative symptoms. The frequent use of MCS such as suppression and rumination may amplify negative affect and interfere with the processing of distressing experiences, potentially reinforcing paranoid ideation or emotional withdrawal. Conversely, the underuse of ACS and PFC strategies, such as cognitive reappraisal or active problem‐solving, may limit opportunities for emotional regulation and adaptive functioning. These findings highlight the importance of targeting coping style in SZ and CHR in therapeutic interventions, particularly by promoting adaptive, problem‐focused strategies and reducing reliance on maladaptive emotional responses.

It is worth noting that certain strategies, such as distraction, are not inherently adaptive or maladaptive. While distraction is often classified as an adaptive coping strategy (Ludwig, Mehl, Krkovic, and Lincoln 2020), its effectiveness depends on the context, duration, and function. For instance, distraction can help regulate short‐term distress or interrupt rumination. However, when used rigidly to avoid emotional processing (in the context of experiential avoidance), it may hinder long‐term emotional adjustment. Distraction combined with *acceptance* has been associated with improved wellbeing, whereas distraction combined with *avoidance* has been associated with poorer outcomes (e.g., lower QoL and higher negative affect) (Wolgast and Lundh 2017). Ultimately, the adaptiveness of distraction hinges not on the strategy itself, but on how and why it is used.

Moreover, the intensity of a stressor not only influences which strategies individuals select but also how effective those strategies are. For example, distraction can be helpful for more intense stressors, while reappraisal tends to be more effective in less intense circumstances (Shafir et al. 2015), but this too depends on the nature of the emotional context. This underscores a broader principle that the adaptiveness of emotional coping strategies depends not solely on their type, but on how, when, and in what way they are used, hence the importance of carrying out a functional analysis of emotional coping in psychosis.

Emotional dysregulation appears to be present in CHR individuals, suggesting it may serve as a potential marker of vulnerability to psychosis. Although most studies in this review were cross‐sectional, the consistent findings of impaired emotional awareness, heightened emotional reactivity, emotional dysregulation, and reliance on maladaptive coping strategies (e.g., suppression and avoidance) in CHR samples suggests these factors may contribute to increased risk of symptom persistence and/or transition. How CHR individuals cope with distressing emotional experiences may influence their clinical trajectory, by either exacerbating vulnerability when regulation fails or by potentially buffering against illness progression when adaptive strategies are employed. These findings therefore highlight the importance of early interventions targeting emotional regulation in CHR populations, not only to alleviate distress but also to potentially modify the course of emerging psychosis.

### 4.1 Strengths and Limitations

This systematic review sought to examine the role of emotions in psychosis onset, symptom persistence, and recovery with the view to facilitating formulation development in CBTp. Employing a mixed methods synthesis of quantitative and qualitative studies enabled a comprehensive synthesis of the extant findings regarding the role of emotions in psychosis, which is a strength of the current review (Sandelowski et al. 2006). From these findings, we have proposed two novel cognitive models, namely the *Emotion Coping Model for Psychosis* and the *Affective Pathway Model of Psychosis*, both of which can be used to enhance CBTp practices.

Some limitations of the present study pertain to the risk of not capturing relevant papers from the search outputs despite a manual search also being conducted. Additionally, the current review only included studies published in English and peer‐reviewed journals, possibly overlooking applicable research such as grey literature. Fifty‐one out of seventy‐eight studies reviewed had sample sizes ranging between 21 and 62, potentially rendering some studies underpowered to detect statistically significant between‐group effects. Emotion was investigated in SZ samples (*n* = 60 studies), CHR (*n* = 15 studies), and in FEP (*n* = 6 studies), yet none of the studies examined a comparison of emotion across CHR, FEP, and SZ to elucidate any emotion related differences between groups. Furthermore, as most studies (*n* = 42) employed cross‐sectional designs, causal inferences cannot be made. For example, while the relationship between emotion regulation and symptoms of SZ is of interest, the studies did not provide insight into the temporal patterns that might explain this relationship. Investigations employing ecological momentary assessment could help address this gap. Longitudinal studies are also needed to clarify how emotional coping strategies influence the development and course of psychosis symptoms, particularly in relation to relapse risk in FEP and the potential for transition to psychosis in CHR individuals.

### 4.2 Clinical Implications

The findings of the present review highlight several important clinical implications regarding the role of emotion in psychosis. SZ demonstrated emotional functioning deficits, stemming from childhood adversities leading to increased emotional reactivity and heightened stress sensitivity. The *Affective Pathway Model of Psychosis* proposed in the present review may therefore aid in devising personalised and idiosyncratic formulations regarding the role of emotions in psychosis within CBTp. Significant differences were found between the implementation of MCS and ACS, with SZ and CHR using the former more. In the recovery process, developing the ability to manage symptoms and fluctuations in the illness's progression is a key goal (Beauchamp et al. 2013). On this point, learning ACS is particularly relevant for recovery (Dunkley 2015). We have therefore developed the *Emotion Coping Model for Psychosis*, a novel cognitive model that underscores the importance of clinicians identifying and targeting MCS and improving ACS. To support clinicians in applying these ideas in practice, we provide Socratic questions and a clinical tool to guide the functional analysis of emotional coping. Clinicians may also draw on components of evidence‐based therapeutic frameworks that target emotion regulation and coping skill development, including Dialectical Behaviour Therapy (DBT) (Lawlor et al. 2022; Neacsiu et al. 2014), Acceptance and Commitment Therapy (ACT) (Spidel et al. 2018; Zoromba et al. 2024), and the Unified Protocol, which is a transdiagnostic treatment for emotional disorders (Barlow et al. 2017). These models offer structured approaches that include chain analysis, acceptance, mindfulness, and emotion regulation strategies, which align with the clinical goals outlined in this review and may be adapted for use with psychosis populations.

### 4.3 Future Directions

Future studies would benefit from the examination of emotion and emotion coping across CHR, FEP, and SZ to elucidate any between‐group differences. It is possible that emotional coping in CHR and FEP is qualitatively different to individuals with advanced chronicity as in SZ who may have developed alternative ways of coping and responding to emotional distress due to their longer illness duration. Understanding these differences could inform the development of stage‐specific interventions. Moreover, while both CHR and FEP populations have been understudied in relation to emotion and emotional coping, research in FEP samples has been particularly limited. Further investigations are needed to better understand how emotions and types of emotional coping may contribute to psychosis onset and symptom persistence. For example, future studies could examine how different forms of distraction (e.g., acceptance‐based vs. avoidance‐based) influence clinical outcomes in psychosis. Additionally, an exploration of meta‐emotional beliefs regarding the perceived dangerousness, uncontrollability, and enduring nature of emotions would aid in devising targeted emotion‐focused interventions for psychosis. In addition, there is a need for interventions that specifically aim to increase PA, given its role in well‐being and functional recovery in psychosis. Future intervention research could also explicitly target specific emotions, negative affect, emotional regulation, and emotional coping to determine their role in preventing transition to psychosis in CHR as well as in preventing relapse and promoting recovery in FEP. There is also a notable lack of qualitative research exploring the role of emotion before, during and after an episode of psychosis. Gaining insight into the subjective experience of emotion and coping across illness stages would offer clinically meaningful data that could enhance the personal relevance and effectiveness of emotion‐focused interventions. Moreover, future research would benefit from examining emotional coping during periods of euthymia, as well as during phases of positive and negative symptoms, to better understand how symptom presence influences coping strategies, and conversely, how emotional coping may shape the course and expression of symptoms. A more nuanced exploration of adaptive versus maladaptive coping strategies within positive symptoms in relation to different hallucinatory subtypes (auditory, visual, tactile, gustatory, olfactory) and delusional subtypes (persecutory, grandiose, somatic), could clarify which coping approaches are most effective or unhelpful for these specific symptom profiles. Such knowledge could then inform more targeted and personalised interventions to improve symptom management and overall functioning. Finally, longitudinal studies with large samples sizes examining how baseline coping strategies predict psychosis symptom severity over time would help clarify their temporal impact on symptom trajectories in psychosis.

## 5 Conclusion

This systematic review highlights the important role emotions play in the manifestation and maintenance of psychosis. Impaired emotional functioning was associated with increased positive and negative symptoms, diminished social functioning and lower QoL. Individuals with SZ exhibited higher levels of NA and lower levels of PA, with NA being a strong predictor of paranoia. The use of MCS, such as suppression, was significantly associated with worsening symptoms and social functioning deficits, while ACS, such as reappraisal, was associated with improved social functioning and negative symptoms. SZ individuals predominantly employed EFC as opposed to PFC, which was associated with negative and positive outcomes, respectively. The *Affective Pathway Model of Psychosis* posits that emotions and emotion coping methods not only contribute to psychosis symptom emergence but also to their persistence and severity. Given the prominence of emotions in the onset and persistence of psychosis symptoms necessitates the development of emotion‐focused interventions for psychosis to not only prevent transition and relapse but to maintain recovery.

## Ethics Statement

The authors have abided by the Ethical Principles of Psychologists and Code of Conduct as set out by the BABCP and BPS.

## Conflicts of Interest

The authors declare no conflicts of interest.

## Data Availability

Data sharing not applicable to this article as no datasets were generated or analysed during the current study.
